# On the Origins of Suboptimality in Human Probabilistic Inference

**DOI:** 10.1371/journal.pcbi.1003661

**Published:** 2014-06-19

**Authors:** Luigi Acerbi, Sethu Vijayakumar, Daniel M. Wolpert

**Affiliations:** 1 Institute of Perception, Action and Behaviour, School of Informatics, University of Edinburgh, Edinburgh, United Kingdom; 2 Doctoral Training Centre in Neuroinformatics and Computational Neuroscience, School of Informatics, University of Edinburgh, Edinburgh, United Kingdom; 3 Computational and Biological Learning Lab, Department of Engineering, University of Cambridge, Cambridge, United Kingdom; Duke University, United States of America

## Abstract

Humans have been shown to combine noisy sensory information with previous experience (priors), in qualitative and sometimes quantitative agreement with the statistically-optimal predictions of Bayesian integration. However, when the prior distribution becomes more complex than a simple Gaussian, such as skewed or bimodal, training takes much longer and performance appears suboptimal. It is unclear whether such suboptimality arises from an imprecise internal representation of the complex prior, or from additional constraints in performing probabilistic computations on complex distributions, even when accurately represented. Here we probe the sources of suboptimality in probabilistic inference using a novel estimation task in which subjects are exposed to an explicitly provided distribution, thereby removing the need to remember the prior. Subjects had to estimate the location of a target given a noisy cue and a visual representation of the prior probability density over locations, which changed on each trial. Different classes of priors were examined (Gaussian, unimodal, bimodal). Subjects' performance was in qualitative agreement with the predictions of Bayesian Decision Theory although generally suboptimal. The degree of suboptimality was modulated by statistical features of the priors but was largely independent of the class of the prior and level of noise in the cue, suggesting that suboptimality in dealing with complex statistical features, such as bimodality, may be due to a problem of acquiring the priors rather than computing with them. We performed a factorial model comparison across a large set of Bayesian observer models to identify additional sources of noise and suboptimality. Our analysis rejects several models of stochastic behavior, including probability matching and sample-averaging strategies. Instead we show that subjects' response variability was mainly driven by a combination of a noisy estimation of the parameters of the priors, and by variability in the decision process, which we represent as a noisy or stochastic posterior.

## Author Summary

The process of decision making involves combining sensory information with statistics collected from prior experience. This combination is more likely to yield ‘statistically optimal’ behavior when our prior experiences conform to a simple and regular pattern. In contrast, if prior experience has complex patterns, we might require more trial-and-error before finding the optimal solution. This partly explains why, for example, a person deciding the appropriate clothes to wear for the weather on a June day in Italy has a higher chance of success than her counterpart in Scotland. Our study uses a novel experimental setup that examines the role of complexity of prior experience on suboptimal decision making. Participants are asked to find a specific target from an array of potential targets given a cue about its location. Importantly, the ‘prior’ information is presented explicitly so that subjects do not need to recall prior events. Participants' performance, albeit suboptimal, was mostly unaffected by the complexity of the prior distributions, suggesting that remembering the patterns of past events constitutes more of a challenge to decision making than manipulating the complex probabilistic information. We introduce a mathematical description that captures the pattern of human responses in our task better than previous accounts.

## Introduction

Humans have been shown to integrate prior knowledge and sensory information in a probabilistic manner to obtain optimal (or nearly so) estimates of behaviorally relevant stimulus quantities, such as speed [1], [2], orientation [3], direction of motion [4], interval duration [5]–[8] and position [9]–[11]. Prior expectations about the values taken by the task-relevant variable are usually assumed to be learned either from statistics of the natural environment [1]–[3] or during the course of the experiment [4]–[6], [8]–[11]; the latter include studies in which a pre-existing prior is modified in the experimental context [12], [13]. Behavior in these perceptual and sensorimotor tasks is qualitatively and often quantitatively well described by Bayesian Decision Theory (BDT) [14], [15].

The extent to which we are capable of performing probabilistic inference on complex distributions that go beyond simple Gaussians, and the algorithms and approximations that we might use, is still unclear [14]. For example, it has been suggested that humans might approximate Bayesian computations by drawing random samples from the posterior distribution [16]–[19]. A major problem in testing hypotheses about human probabilistic inference is the difficulty in identifying the source of suboptimality, that is, separating any constraints and idiosyncrasies in performing Bayesian computations per se from any deficiencies in learning and recalling the correct prior. For example, previous work has examined Bayesian integration in the presence of experimentally-imposed bimodal priors [4], [8], [9], [20]. Here the normative prescription of BDT under a wide variety of assumptions would be that responses should be biased towards one peak of the distribution or the other, depending on the current sensory information. However, for such bimodal priors, the emergence of Bayesian biases can require thousands of trials [9] or be apparent only on pooled data [4], and often data show at best a complex pattern of biases which is only in partial agreement with the underlying distribution [8], [20]. It is unknown whether this mismatch is due to the difficulty of learning statistical features of the bimodal distribution or if the bimodal prior is actually fully learned but our ability to perform Bayesian computation with it is limited. In the current study we look systematically at how people integrate uncertain cues with trial-dependent ‘prior’ distributions that are explicitly made available to the subjects. The priors were displayed as an array of potential targets distributed according to various density classes – Gaussian, unimodal or bimodal. Our paradigm allows full control over the generative model of the task and separates the aspect of computing with a probability distribution from the problem of learning and recalling a prior. We examine subjects' performance in manipulating probabilistic information as a function of the shape of the prior. Participants' behavior in the task is in qualitative agreement with Bayesian integration, although quite variable and generally suboptimal, but the degree of suboptimality does not differ significantly across different classes of distributions or levels of reliability of the cue. In particular, performance was not greatly affected by complexity of the distribution per se – for instance, people's performance with bimodal priors is analogous to that with Gaussian priors, in contrast to previous learning experiments [8], [9]. This finding suggests that major deviations encountered in previous studies are likely to be primarily caused by the difficulty in learning complex statistical features rather than computing with them.

We systematically explore the sources of suboptimality and variability in subjects' responses by employing a methodology that has been recently called *factorial model comparison* [21]. Using this approach we generate a set of models by combining different sources of suboptimality, such as different approximations in decision making with different forms of sensory noise, in a factorial manner. Our model comparison is able to reject some common models of variability in decision making, such as probability matching with the posterior distribution (posterior-matching) or a sampling-average strategy consisting of averaging a number of samples from the posterior distribution. The observer model that best describes the data is a Bayesian observer with a slightly mismatched representation of the likelihoods, with sensory noise in the estimation of the parameters of the prior, that occasionally lapses, and most importantly has a stochastic representation of the posterior that may represent additional variability in the inference process or in action selection.

## Results

**Figure 1:**
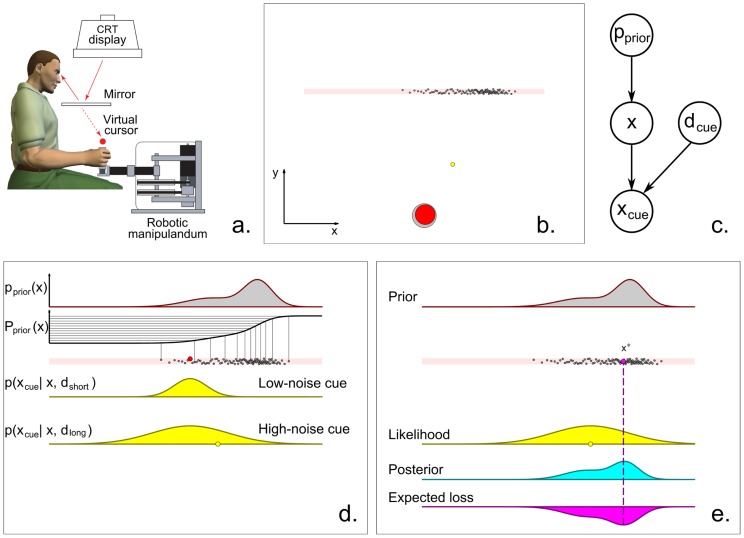
Experimental procedure. **a: Setup.** Subjects held the handle of a robotic manipulandum. The visual scene from a CRT monitor, including a cursor that tracked the hand position, was projected into the plane of the hand via a mirror. **b: Screen setup.** The screen showed a home position (grey circle), the cursor (red circle) here at the start of a trial, a line of potential targets (dots) and a visual cue (yellow dot). The task consisted in locating the true target among the array of potential targets, given the position of the noisy cue. The coordinate axis was not displayed on screen, and the target line is shaded here only for visualization purposes. **c: Generative model of the task.** On each trial the position of the hidden target 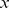 was drawn from a discrete representation of the trial-dependent prior 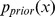, whose shape was chosen randomly from a session-dependent class of distributions. The vertical distance of the cue from the target line, 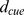, was either ‘short’ or ‘long’, with equal probability. The horizontal position of the cue, 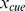, depended on 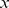 and 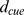. The participants had to infer 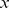 given 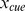, 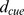 and the current prior 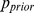. **d: Details of the generative model.** The potential targets constituted a discrete representation of the trial-dependent prior distribution 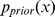; the discrete representation was built by taking equally spaced samples from the inverse of the cdf of the prior, 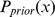. The true target (red dot) was chosen uniformly at random from the potential targets, and the horizontal position of the cue (yellow dot) was drawn from a Gaussian distribution, 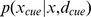, centered on the true target 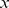 and whose SD was proportional to the distance 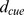 from the target line (either ‘short’ or ‘long’, depending on the trial, for respectively low-noise and high-noise cues). Here we show the location of the cue for a high-noise trial. **e: Components of Bayesian decision making.** According to Bayesian Decision Theory, a Bayesian ideal observer combines the prior distribution with the likelihood function to obtain a posterior distribution. The posterior is then convolved with the loss function (in this case whether the target will be encircled by the cursor) and the observer picks the ‘optimal’ target location 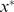 (purple dot) that corresponds to the minimum of the expected loss (dashed line).

Subjects were required to locate an unknown target given probabilistic information about its position along a target line (Figure 1a–b). Information consisted of a visual representation of the a priori probability distribution of targets for that trial and a noisy cue about the actual target position (Figure 1b). On each trial a hundred potential targets (dots) were displayed on a horizontal line according to a discrete representation of a trial-dependent ‘prior’ distribution 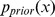. The true target, unknown to the subject, was chosen at random from the potential targets with uniform probability. A noisy cue with horizontal position 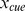, drawn from a normal distribution centered on the true target, provided partial information about target location. The cue had distance 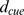 from the target line, which could be either a short distance, corresponding to added noise with low-variance, or a long distance, with high-variance noise. Both prior distribution and cue remained on screen for the duration of the trial. (See Figure 1c–d for the generative model of the task.) The task for the subjects involved moving a circular cursor controlled by a manipulandum towards the target line, ending the movement at their best estimate for the position of the real target. A ‘success’ ensued if the true target was within the cursor radius.

To explain the task, subjects were told that the each dot represented a child standing in a line in a courtyard, seen from a bird's eye view. On each trial a random child was chosen and, while the subject was ‘not looking’, the child threw a yellow ball (the cue) directly ahead of them towards the opposite wall. Due to their poor throwing skills, the farther they threw the ball the more imprecise they were in terms of landing the ball straight in front of them. The subject's task was to identify the child who threw the ball, after seeing the landing point of the ball, by encircling him or her with the cursor. Subjects were told that the child throwing the ball could be any of the children, chosen randomly each trial with equal probability.

**Figure 2:**
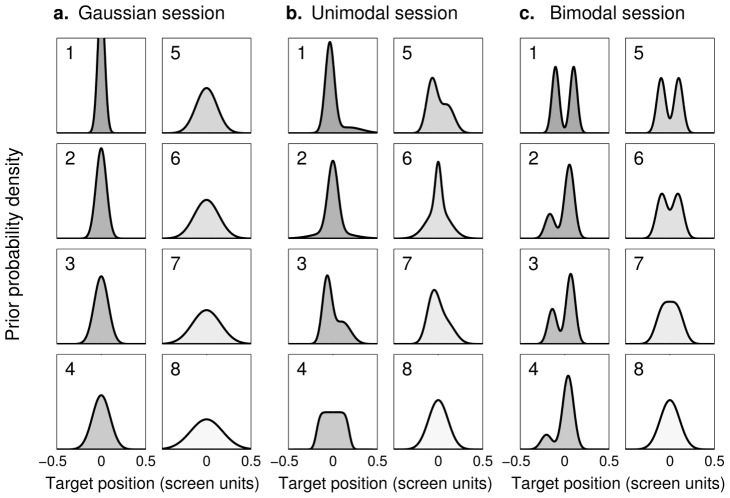
Prior distributions. Each panel shows the (unnormalized) probability density for a ‘prior’ distribution of targets, grouped by experimental session, with eight different priors per session. Within each session, priors are numbered in order of increasing differential entropy (i.e. increasing variance for Gaussian distributions). During the experiment, priors had a random location (mean drawn uniformly) and asymmetrical priors had probability 1/2 of being ‘flipped’. Target positions are shown in standardized screen units (from 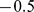 to 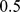). **a: Gaussian priors.** These priors were used for the training session, common to all subjects, and in the Gaussian test session. Standard deviations cover the range 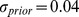 to 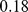 screen units in equal increments. **b: Unimodal priors.** All unimodal priors have fixed SD 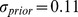 screen units but different skewness and kurtosis (see Methods for details). **c: Bimodal priors.** All priors in the bimodal session have fixed SD 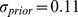 screen units but different relative weights and separation between the peaks (see Methods).

Twenty-four subjects performed a training session in which the ‘prior’ distributions of targets shown on the screen (the set of children) corresponded to Gaussian distributions with a standard deviation (SD) that varied between trials (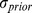 from 0.04 to 0.18 standardized screen units; Figure 2a). On each trial the location (mean) of the prior was chosen randomly from a uniform distribution. Half of the trials provided the subjects with a ‘short-distance’ cue about the position of the target (low noise: 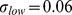 screen units; a short throw of the ball); the other half had a ‘long-distance’ cue (high noise: 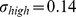 screen units; a long throw). The actual position of the target (the ‘child’ who threw the ball) was revealed at the end of each trial and a displayed score kept track of the number of ‘successes’ in the session (full performance feedback). The training session allowed subjects to learn the structure of the task in a setting in which humans are known to perform in qualitative and often quantitative agreement with Bayesian Decision Theory, i.e. under Gaussian priors [5], [9]–[11]. Note however that, in contrast with the previous studies, our subjects were required to compute each trial with a different Gaussian distribution (Figure 2a). The use of Gaussian priors in the training session allowed us to assess whether our subjects could use explicit priors in our novel experimental setup in the same way in which they have been shown to learn Gaussian priors through extended implicit practice.

After the training session, subjects were randomly divided in three groups (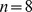 each) to perform a test session. Test sessions differed with respect to the class of prior distributions displayed during the session. For the ‘Gaussian test’ group, the distributions were the same eight Gaussian distributions of varying SD used during training (Figure 2a). For the ‘unimodal test’ group, on each trial the prior was randomly chosen from eight unimodal distributions with fixed SD (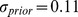 screen units) but with varying skewness and kurtosis (see Methods and Figure 2b). For the ‘bimodal test’ group, priors were chosen from eight (mostly) bimodal distributions with fixed SD (again, 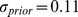 screen units) but variable separation and weighting between peaks (see Methods and Figure 2c). As in the training session, on each trial the mean of the prior was drawn randomly from a uniform distribution. To preserve global symmetry during the session, asymmetric priors were ‘flipped’ along their center of mass with a probability of 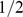. During the test session, at the end of each trial subjects were informed whether they ‘succeeded’ or ‘missed’ the target but the target's actual location was not displayed (partial feedback). The ‘Gaussian test’ group allowed us to verify that subjects’ behavior would not change after removal of full performance feedback. The ‘unimodal test’ and ‘bimodal test’ groups provided us with novel information on how subjects perform probabilistic inference with complex distributions. Moreover, non-Gaussian priors allowed us to evaluate several hypotheses about subjects’ behavior that are not testable with Gaussian distributions alone [22].

### Human performance

**Figure 3:**
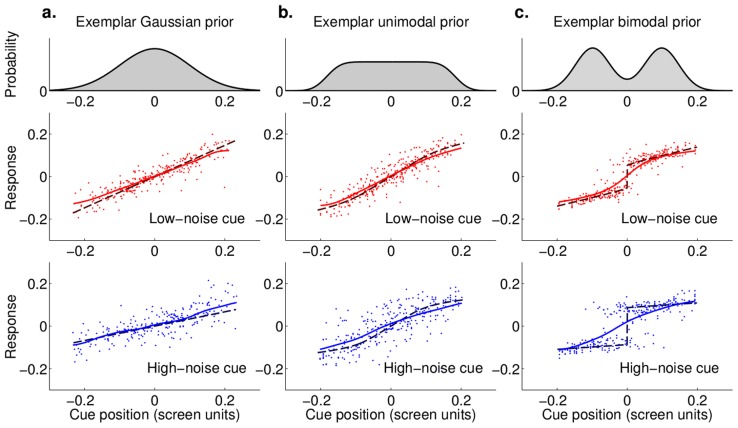
Subjects' responses as a function of the position of the cue. Each panel shows the pooled subjects' responses as a function of the position of the cue either for low-noise cues (red dots) or high-noise cues (blue dots). Each column corresponds to a representative prior distribution, shown at the top, for each different group (Gaussian, unimodal and bimodal). In the response plots, dashed lines correspond to the Bayes optimal strategy given the generative model of the task. The continuous lines are a kernel regression estimate of the mean response (see Methods). **a**. Exemplar Gaussian prior (prior 4 in Figure 2a). **b**. Exemplar unimodal prior (platykurtic distribution: prior 4 in Figure 2b). **c**. Exemplar bimodal prior (prior 5 in Figure 2c). Note that in this case the mean response is not necessarily a good description of subjects' behavior, since the marginal distribution of responses for central positions of the cue is bimodal.

We first performed a model-free analysis of subjects' performance. Figure 3 shows three representative prior distributions and the pooled subjects' responses as a function of the cue position for low (red) and high (blue) noise cues. Note that pooled data are used here only for display and all subjects' datasets were analyzed individually. The cue positions and responses in Figure 3 are reported in a coordinate system relative to the mean of the prior (set as 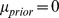). For all analyses we consider relative coordinates without loss of generality, having verified the assumption of translational invariance of our task (see Section 1 in Text S1).

Figure 3 shows that subjects' performance was affected by both details of the prior distribution and the cue. Also, subjects' mean performance (continuous lines in Figure 3) show deviations from the prediction of an optimal Bayesian observer (dashed lines), suggesting that subjects behavior may have been suboptimal.

#### Linear integration with Gaussian priors

We examined how subjects performed in the task under the well-studied case of Gaussian priors [9], [10]. Given a Gaussian prior with SD 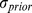 and a noisy cue with horizontal position 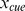 and known variability 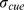 (assuming Gaussian noise), the most likely target location can be computed through Bayes' theorem. In the relative coordinate system (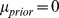), the optimal target location takes the simple linear form: 
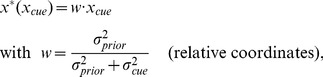
(1)where 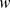 is the linear weight assigned to the cue.

**Figure 4:**
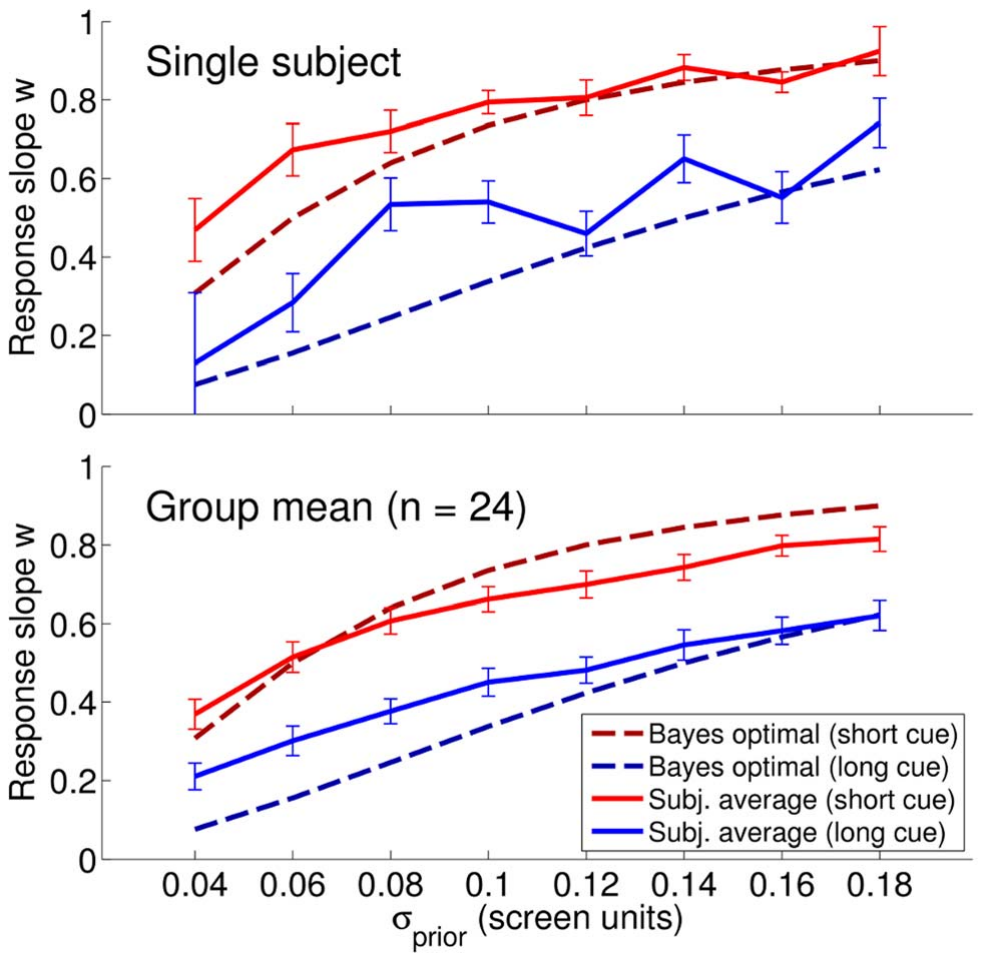
Response slopes for the training session. Response slope 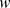 as a function of the SD of the Gaussian prior distribution, 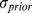, plotted respectively for trials with low noise (‘short’ cues, red line) and high noise (‘long’ cues, blue line). The response slope is equivalent to the linear weight assigned to the position of the cue (Eq. 1). Dashed lines represent the Bayes optimal strategy given the generative model of the task in the two noise conditions. *Top*: Slopes for a representative subject in the training session (slope 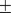 SE). *Bottom*: Average slopes across all subjects in the training session (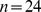, mean 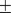 SE across subjects).

We compared subjects' behavior with the ‘optimal’ strategy predicted by Eq. 1 (see for instance Figure 3a; the dashed line corresponds to the optimal strategy). For each subject and each combination of 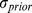 and cue type (either ‘short’ or ‘long’, corresponding respectively to low-noise and high-noise cues), we fit the responses 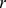 as a function of the cue position 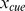 with a robust linear fit. The slopes of these fits for the training session are plotted in Figure 4; results were similar for the Gaussian test session. Statistical differences between different conditions were assessed using repeated-measures ANOVA (rm-ANOVA) with Greenhouse-Geisser correction (see Methods).

In general, subjects did not perform exactly as predicted by the optimal strategy (dashed lines), but they took into account the probabilistic nature of the task. Specifically, subjects tended to give more weight to low-noise cues than to high-noise ones (main effect: Low-noise cues, High-noise cues; 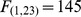, 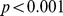), and the weights were modulated by the width of the prior (main effect: prior width 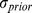; 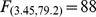, 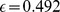, 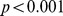), with wider priors inducing higher weighting of the cue. Interestingly, cue type and width of the prior seemed to influence the weights independently, as no significant interaction was found (interaction: prior width 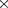 cue type; 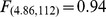, 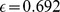, 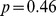). Analogous patterns were found in the Gaussian test session.

We also examined the average bias of subjects' responses (intercept of linear fits), which is expected to be zero for the optimal strategy. On average subjects exhibited a small but significant rightward bias in the training session of 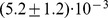 screen units or 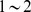 mm (mean 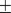 SE across subjects, 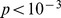). The average bias was only marginally different than zero in the test session: 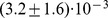 screen units (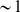 mm, 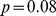).

#### Optimality index

**Figure 5:**
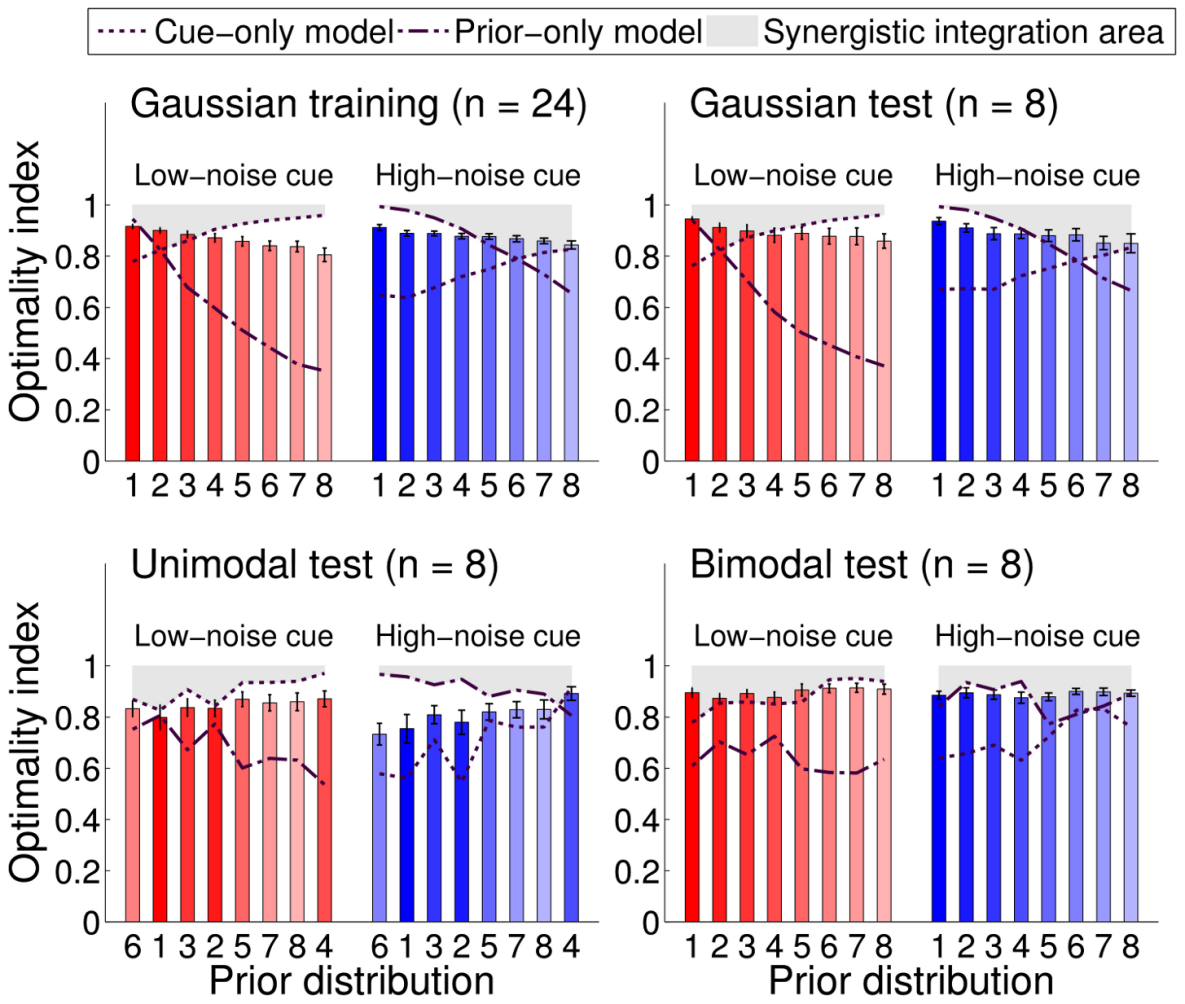
Group mean optimality index. Each bar represents the group-averaged optimality index for a specific session, for each prior (indexed from 1 to 8, see also Figure 2) and cue type, low-noise cues (red bars) or high-noise cues (blue bars). The optimality index in each trial is computed as the probability of locating the correct target based on the subjects' responses divided by the probability of locating the target for an optimal responder. The maximal optimality index is 1, for a Bayesian observer with correct internal model of the task and no sensorimotor noise. Error bars are SE across subjects. Priors are arranged in the order of differential entropy (i.e. increasing variance for Gaussian priors), except for ‘unimodal test’ priors which are listed in order of increasing width of the main peak in the prior (see text). The dotted line and dash-dotted line represent the optimality index of a suboptimal observer that takes into account respectively either only the cue or only the prior. The shaded area is the zone of synergistic integration, in which an observer performs better than using information from either the prior or the cue alone.

We developed a general measure of performance that is applicable beyond the Gaussian case. An objective measure of performance in each trial is the success probability, that is, the probability that the target would be within a cursor radius' distance from the given response (final position of the cursor) under the generative model of the task (see Methods). We defined the *optimality index* for a trial as the success probability normalized by the maximal success probability (the success probability of an optimal response). The optimality index allows us to study variations in subjects' performance which are not trivially induced by variations in the difficulty of the task. Figure 5 shows the optimality index averaged across subjects for different conditions, in different sessions. Data are also summarized in Table 1. Priors in Figure 5 are listed in order of differential entropy (which corresponds to increasing variance for Gaussian priors), with the exception of ‘unimodal test’ priors which are in order of increasing width of the main peak in the prior, as computed through a Laplace approximation. We chose this ordering for priors in the unimodal test session as it highlights the pattern in subjects' performance (see below).

**Table 1:** Group mean optimality index.

Session	Low-noise cue	High-noise cue	All cues
Gaussian training	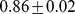	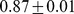	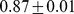
Gaussian test	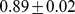	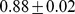	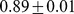
Unimodal test	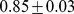	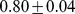	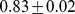
Bimodal test	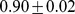	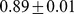	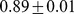
All sessions	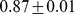	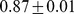	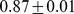

Each entry reports mean 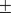 SE of the group optimality index for a specific session and cue type, or averaged across all sessions/cues. See also Figure 5.

For a comparison, Figure 5 also shows the optimality index of two suboptimal models that represent two extremal response strategies. Dash-dotted lines correspond to the optimality index of a Bayesian observer that maximizes the probability of locating the correct target considering only the prior distribution (see below for details). Conversely, dotted lines correspond to an observer that only uses the cue and ignores the prior: that is, the observer's response in a trial matches the current position of the cue. The shaded gray area specifies the ‘synergistic integration’ zone, in which the subject is integrating information from both prior and cue in a way that leads to better performance than by using either the prior or the cue alone. Qualitatively, the behavior in the gray area can be regarded as ‘close to optimal’, whereas performance below the gray area is suboptimal. As it is clear from Figure 5, in all sessions participants were sensitive to probabilistic information from both prior and cue – that is, performance is always above the minimum of the extremal models (dash-dotted and dotted lines) – in agreement with what we observed in Figure 4 for Gaussian sessions, although their integration was generally suboptimal. Human subjects were analogously found to be suboptimal in a previous task that required to take into account explicit probabilistic information [23].

We examined how the optimality index changed across different conditions. From the analysis of the training session, it seems that subjects were able to integrate low-noise and high-noise cues for priors of any width equally well, as we found no effect of cue type on performance (main effect: Low-noise cues, High-noise cues; 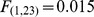, 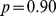) and no significant interaction between cue types and prior width (interaction: prior width 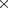 cue type; 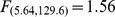, 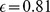, 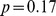). However, relative performance was significantly affected by the width of the prior per se (main effect: prior width 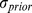; 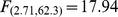, 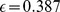, 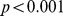); people tended to perform worse with wider priors, in a way that is not simply explained by the objective decrease in the probability of locating the correct target due to the less available information (see Discussion).

Results in the Gaussian test session (Figure 5 top right) replicated what we had obtained in the training session. Subjects' performance was not influenced by cue type (main effect: Low-noise cues, High-noise cues; 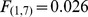, 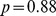) nor by the interaction between cue types and prior width (interaction: prior width 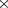 cue type; 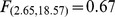, 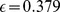, 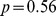). Conversely, as before, the width of the prior affected performance significantly (main effect: prior width 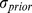; 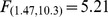, 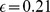, 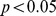); again, wider priors were associated with lower relative performance.

A similar pattern of results was found also for the bimodal test session (Figure 5 bottom right). Performance was affected significantly by the shape of the prior (main effect: prior shape; 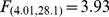, 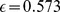, 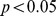) but otherwise participants integrated cues of different type with equal skill (main effect: Low-noise cues, High-noise cues; 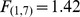, 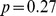; interaction: prior shape 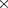 cue type; 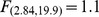, 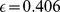, 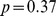). However, in this case performance was not clearly correlated with a simple measure of the prior or of the average posterior (e.g. differential entropy).

Another scenario emerged in the unimodal test session (Figure 5 bottom left). Here, subjects' performance was affected not only by the shape of the prior (main effect: prior shape; 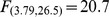, 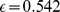, 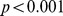) but also by the type of cue (main effect: Low-noise cues, High-noise cues; 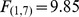, 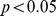) and the specific combination of cue and prior (interaction: prior shape 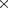 cue type; 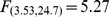, 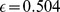, 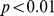). Moreover, in this session performance improved for priors whose main peak was broader (see Discussion).

Notwithstanding this heterogeneity of results, an overall comparison of participants' relative performance in test sessions (averaging results over priors) did not show statistically significant differences between groups (main effect: group; 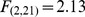, 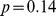) nor between the two levels of reliability of the cue (main effect: Low-noise cues, High-noise cues; 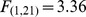, 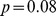); only performance in the unimodal session for high-noise cues was at most marginally worse. In particular, relative performance in the Gaussian test and the bimodal test sessions was surprisingly similar, unlike previous learning experiments (see Discussion).

#### Effects of uncertainty on reaction time

**Figure 6:**
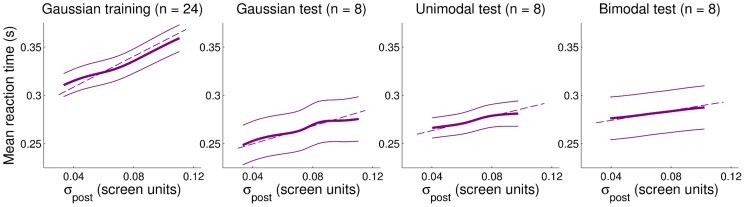
Average reaction times as a function of the SD of the posterior distribution. Each panel shows the average reaction times (mean 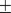 SE across subjects) for a given session as a function of the SD of the posterior distribution, 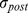 (individual data were smoothed with a kernel regression estimate, see Methods). Dashed lines are robust linear fits to the reaction times data. For all sessions the slope of the linear regression is significantly different than zero (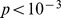).

Lastly, we examined the effect of uncertainty on subjects' reaction time (time to start movement after the ‘go’ beep) in each trial. Uncertainty was quantified as the SD of the posterior distribution in the current trial, 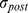 (an alternative measure of spread, exponential entropy [24], gave analogous results). We found that the average subjects' reaction time grew almost linearly with 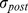 (Figure 6). The average change in reaction times (from lowest to highest uncertainty in the posterior) was substantial during the training session (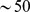 ms, about 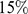 change), although less so in subsequent test sessions.

### Suboptimal Bayesian observer models

Our model-free analysis showed that subjects' performance in the task was suboptimal. Here we examine the source of this apparent suboptimality. Subjects' performance is modelled with a family of Bayesian ideal observers which incorporate various hypotheses about the decision-making process and internal representation of the task, with the aim of teasing out the major sources of subjects' suboptimality; see Figure 1e for a depiction of the elements of decision making in a trial. All these observers are ‘Bayesian’ because they build a posterior distribution through Bayes' rule, but the operations they perform with the posterior can differ from the normative prescriptions of Bayesian Decision Theory (BDT).

We construct a large model set with a factorial approach that consists in combining different independent model ‘factors’ that can take different ‘levels’ [8], [21]. The basic factors we consider are:

1. *Decision making* (3 levels): Bayesian Decision Theory (‘BDT’), stochastic posterior (‘SPK’), posterior probability matching (‘PPM’).
2. *Cue-estimation sensory noise* (2 levels): absent or present (‘S’).
3. *Noisy estimation of the prior* (2 levels): absent or present (‘P’).
4. *Lapse* (2 levels): absent or present (‘L’).

Observer models are identified by a model string, for example ‘BDT-P-L’ indicates an observer model that follows BDT with a noisy estimate of the prior and suffers from occasional lapses. Our basic model set comprises 24 observer models; we also considered several variants of these models that are described in the text. All main factors are explained in the following sections and summarized in Table 2. The term ‘model component' is used through the text to indicate both factors and levels.

**Table 2:** Set of model factors.

Label	Model description	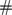 parameters	Free parameters ( 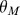 )
BDT	Decision making: BDT	4	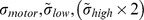
PPM	Decision making: Posterior probability matching	4	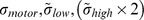
SPK	Decision making: Stochastic posterior	6	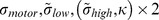
PSA	Decision making: Posterior sampling average (^*^)	6	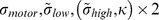
S	Cue-estimation noise	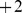	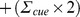
P	Prior estimation noise	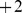	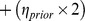
L	Lapse	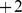	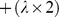
MV	Gaussian approximation: mean/variance (^*^)	–	–
LA	Gaussian approximation: Laplace approximation (^*^)	–	–

Table of all major model factors, identified by a label and short description. An observer model is built by choosing a model level for decision making and then optionally adding other components. For each model component the number of free parameters is specified. A ‘
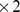
’ means that a parameter is specified independently for training and test sessions; otherwise parameters are shared across sessions. See main text and Methods for the meaning of the various parameters. (^*^) These additional components appear in the comparison of alternative models of decision making.

#### Decision making: Standard BDT observer (‘BDT’)

The ‘decision-making’ factor comprises model components with different assumptions about the decision process. We start describing the ‘baseline’ Bayesian observer model, BDT, that follows standard BDT. Suboptimality, in this case, emerges if the observer’s internal estimates of the parameters of the task take different values from the true ones. As all subsequent models are variations of the BDT observer we describe this model in some detail.

On each trial the information available to the observer is comprised of the ‘prior’ distribution 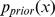, the cue position 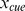, and the distance 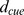 of the cue from the target line, which is a proxy for cue variability, 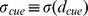. The posterior distribution of target location, 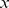, is computed by multiplying together the prior with the likelihood function. For the moment we assume the observer has perfect access to the displayed cue location and prior, and knowledge that cue variability is normally distributed. However, we allow the observer's estimate of the variance of the likelihood (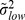 and 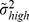) to mismatch the actual variance (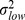 and 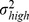). Therefore the posterior is given by: 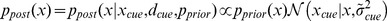
(2)where 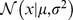 denotes a normal distribution with mean 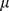 and variance 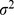.

In general, for any given trial, the choice the subject makes (desired pointing location for 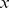) can be a probabilistic one, leading to a ‘target choice’ distribution. However, for standard BDT, the choice is deterministic given the trial parameters, leading to a ‘target choice’ distribution that collapses to a delta function: 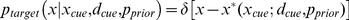
(3)where 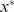 is the ‘optimal’ target position that minimizes the observer's expected loss. The explicit task in our experiment is to place the target within the radius of the cursor, which is equivalent to a ‘square well’ loss function with a window size equal to the diameter of the cursor. For computational reasons, in our observer models we approximate the square well loss with an inverted Gaussian (see Methods) that best approximates the square well, with fixed SD 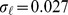 screen units (see Section 3 in Text S1).

In our experiment all priors were mixtures of 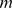 (mainly 1 or 2) Gaussian distributions of the form 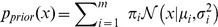, with 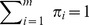. It follows that the expected loss is a mixture of Gaussians itself, and the optimal target that minimizes the expected loss takes the form (see Methods for details): 
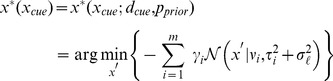
(4)where we defined:



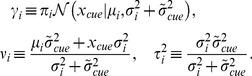
(5)


For a single-Gaussian prior (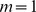), 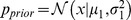 and the posterior distribution is itself a Gaussian distribution with mean 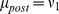 and variance 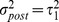, so that 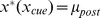.

We assume that the subject's response is corrupted by motor noise, which we take to be normally distributed with SD 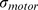. By convolving the target choice distribution (Eq. 3) with motor noise we obtain the final response distribution: 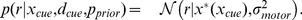
(6)


The calculation of the expected loss in Eq. 4 does not explicitly take into account the consequences of motor variability, but this approximation has minimal effects on the inference (see Discussion).

The behavior of observer model BDT is completely described by Eqs. 4, 5 and 6. This observer model is *subjectively* Bayes optimal; the subject applies BDT to his or her internal model of the task, which might be wrong. Specifically, the observer will be close to *objective* optimality only if his or her estimates for the likelihood parameters, 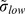 and 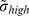, match the true likelihood parameters of the task (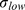 and 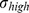). As extreme cases, if 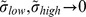 the BDT observer will ignore the prior and only use the noiseless cues (cue-only observer model; dashed lines in Figure 5), whereas for 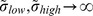 the observer will use only probabilistic information contained in the priors (prior-only observer model; dotted lines in Figure 5).

#### Decision making: Noisy decision makers (‘SPK’ and ‘PPM’)

An alternative to BDT is a family of observer models in which the decision-making process is probabilistic, either because of noise in the inference or stochasticity in action selection. We model these various sources of variability without distinction as stochastic computations that involve the posterior distribution.

**Figure 7:**
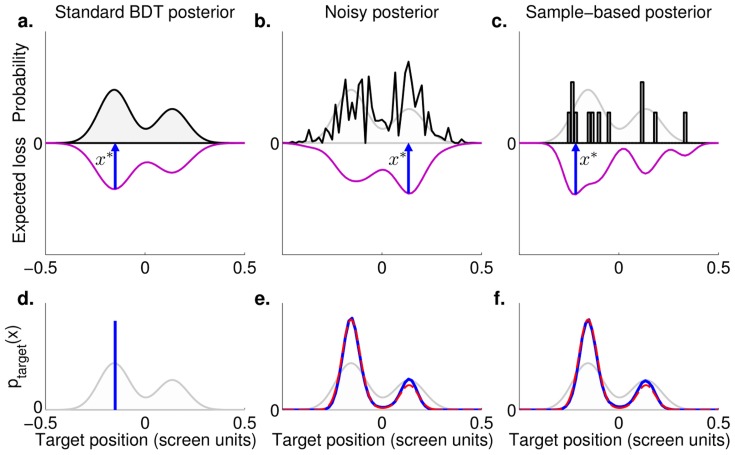
Decision making with stochastic posterior distributions. **a–c**: Each panel shows an example of how different models of stochasticity in the representation of the posterior distribution, and therefore in the computation of the expected loss, may affect decision making in a trial. In all cases, the observer chooses the subjectively optimal target 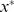 (blue arrow) that minimizes the expected loss (purple line; see Eq. 4) given his or her current representation of the posterior (black lines or bars). The original posterior distribution is showed in panels b–f for comparison (shaded line). **a**: Original posterior distribution. **b**: Noisy posterior: the original posterior is corrupted by random multiplicative or Poisson-like noise (in this example, the noise has caused the observer to aim for the wrong peak). **c**: Sample-based posterior: a discrete approximation of the posterior is built by drawing samples from the original posterior (grey bars; samples are binned for visualization purposes). **d–f**: Each panel shows how stochasticity in the posterior affects the distribution of target choices 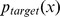 (blue line). **d**: Without noise, the target choice distribution is a delta function peaked on the minimum of the expected loss, as per standard BDT. **e**: On each trial, the posterior is corrupted by different instances of noise, inducing a distribution of possible target choices 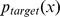 (blue line). In our task, this distribution of target choices is very well approximated by a power function of the posterior distribution, Eq. 7 (red dashed line); see Text S2 for details. **f**: Similarly, the target choice distribution induced by sampling (blue line) is fit very well by a power function of the posterior (red dashed line). Note the extremely close resemblance of panels e and f (the exponent of the power function is the same).

We start our analysis by considering a specific model, SPK (stochastic posterior, 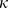-power), in which the observer minimizes the expected loss (Eq. 4) under a noisy, approximate representation of the posterior distribution, as opposed to the deterministic, exact posterior of BDT (Figure 7a and 7d); later we will consider other variants of stochastic computations. As before, we allow the SD of the likelihoods, 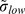 and 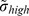, to mismatch their true values. For mathematical and computational tractability, we do not directly simulate the noisy inference during the model comparison. Instead, we showed that different ways of introducing stochasticity in the inference process – either by adding noise to an explicit representation of the observer's posterior (Figure 7b and 7e), or by building a discrete approximation of the posterior via sampling (Figure 7c and 7f) – induce variability in the target choice that is well approximated by a power function of the posterior distribution itself; see Text S2 for details.

We, therefore, use the power function approximation with power 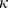 – hence the name of the model – to simulate the effects of a stochastic posterior on decision making, without committing to a specific interpretation. The target choice distribution in model SPK takes the form: 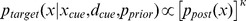
(7)where the power exponent 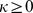 is a free parameter inversely related to the amount of variability. Eq. 7 is convolved with motor noise to give the response distribution. The power function conveniently interpolates between a posterior-matching strategy (for 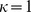) and a maximum a posteriori (MAP) solution (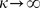).

We consider as a separate factor the specific case in which the power exponent 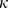 is fixed to 1, yielding a posterior probability matching observer, PPM, that takes action according to a single draw from the posterior distribution [25], [26].

#### Observer models with cue-estimation sensory noise (‘S’)

We consider a family of observer models, S, in which we drop the assumption that the observer perfectly knows the horizontal position of the cue. We model sensory variability by adding Gaussian noise to the internal measurement of 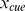, which we label 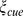: 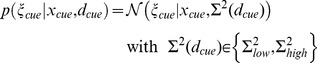
(8)where 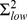, 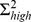 represent the variances of the estimates of the position of the cue, respectively for low-noise (short-distance) and high-noise (long-distance) cues. According to Weber's law, we assume that the measurement error is proportional to the distance from the target line 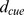, so that the ratio of 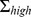 to 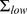 is equal to the ratio of 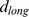 to 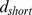, and we need to specify only one of the two parameters (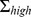). Given that both the cue variability and the observer's measurement variability are normally distributed, their combined variability will still appear to the observer as a Gaussian distribution with variance 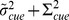, assuming independence. Therefore, the observer's internal model of the task is formally identical to the description we gave before by replacing 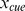 with 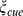 in Eq. 2 (see Methods). Since the subject's internal measurement is not accessible during the experiment, the observed response probability is integrated over the hidden variable 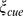 (Eq. 18 in Methods). A model with cue-estimation sensory noise (‘S’) tends to the equivalent observer model without noise for 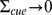.

#### Observer models with noisy estimation of the prior (‘P’)

We introduce a family of observer models, P, in which subjects have access only to noisy estimates of the parameters of the prior, 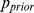. For this class of models we assume that estimation noise is structured along a task-relevant dimension.

Specifically, for Gaussian priors we assume that the observers take a noisy internal measurement of the SD of the prior, 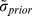, which according to Weber's law follows a log-normal distribution: 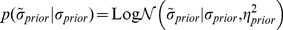
(9)where 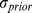, the true SD, is the log-scale parameter and 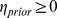 is the shape parameter of the log-normally distributed measurement (respectively mean and SD in log space). We assume an analogous form of noise on the width of the platykurtic prior in the unimodal session. Conversely, we assume that for priors that are mixtures of two Gaussians the main source of error stems from assessing the relative importance of the two components. In this case we add log-normal noise to the weights of each component, which we assume to be estimated independently:
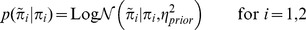
(10)where 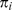 are the true mixing weights and 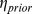 is the noise parameter previously defined. Note that Eq. 10 is equivalent to adding normal noise with SD 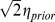 to the log weights ratio in the ‘natural’ log odds space [27].

The internal measurements of 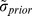 (or 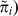 are used by the observer in place of the true parameters of the priors in the inference process (e.g. Eq. 5). Since we cannot measure the internal measurements of the subjects, the actual response probabilities are computed by integrating over the unobserved values of 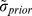 or 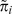 (see Methods). Note that for 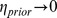 an observer model with prior noise (‘P’) tends to its corresponding version with no noise.

A different type of measurement noise on the the prior density is represented by ‘unstructured’, pointwise noise which can be shown to be indistinguishable from noise in the posterior under certain assumptions (see Text S2).

#### Observer models with lapse (‘L’)

It is possible that the response variability exhibited by the subjects could be simply explained by occasional lapses. Observer models with a lapse term are common in psychophysics to account for missed stimuli and additional variability in the data [28]. According to these models, in each trial the observer has a typically small, fixed probability 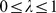 (the *lapse rate*) of making a choice from a lapse probability distribution instead of the optimal target 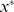. As a representative lapse distribution we choose the prior distribution (prior-matching lapse). The target choice for an observer with lapse has distribution: 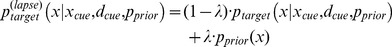
(11)where the first term in the right hand side of the equation is the target choice distribution (either Eq. 3 or Eq. 7, depending on the decision-making factor), weighted by the probability of *not* making a lapse, 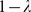. The second term is the lapse term, with probability 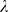, and it is clear that the observer model with lapse (‘L’) reduces to an observer with no lapse in the limit 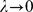. Eq. 11 is then convolved with motor noise to provide the response distribution. We also tested a lapse model in which the lapse distribution was uniform over the range of the displayed prior distribution. Observer models with uniform lapse performed consistently worse than the prior-matching lapse model, so we only report the results of the latter.

### Model comparison

For each observer model 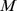 and each subject’s dataset we evaluated the posterior distribution of parameters 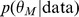, where 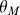 is in general a vector of model-dependent parameters (see Table 2). Each subject's dataset comprised of two sessions (training and test), for a total of about 1200 trials divided in 32 distinct conditions (8 priors 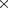 2 noise levels 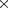 2 sessions). In general, we assumed subjects shared the motor parameter 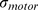 across sessions. We also assumed that from training to test sessions people would use the same high-noise to low-noise ratio between cue variability (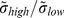); so only one cue-noise parameter (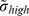) needed to be specified for the test session. Conversely, we assumed that the other noise-related parameters, if present (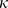, 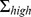, 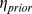, 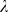), could change freely between sessions, reasoning that additional response variability can be affected by the presence or absence of feedback, or as a result of the difference between training and test distributions. These assumptions were validated via a preliminary model comparison (see Section 5 in Text S1). Table 2 lists a summary of observer models and their free parameters.

The posterior distributions of the parameters were obtained through a slice sampling Monte Carlo method [29]. In general, we assumed noninformative priors over the parameters except for motor noise parameter 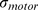 and cue-estimation sensory noise parameter 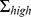 (when present), for which we determined a reasonable range of values through an independent experiment (see Methods and Text S3). Via sampling we also computed for each dataset a measure of complexity and goodness of fit of each observer model, the Deviance Information Criterion (DIC) [30], which we used as an approximation of the marginal likelihood to perform model comparison (see Methods).

We compared observer models according to a hierarchical Bayesian model selection (BMS) method that treats subjects and models as random effects [31]. That is, we assumed that multiple observer models could be present in the population, and we computed how likely it is that a specific model (or model level within a factor) generated the data of a randomly chosen subject, given the model evidence represented by the subjects' DIC scores (see Methods for details). As a Bayesian metric of significance we used the exceedance probability 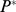 of one model (or model level) being more likely than any other model (or model levels within a factor). In Text S1 we report instead a classical (frequentist) analysis of the group difference in DIC between models (GDIC), which assumes that all datasets have been generated by the same unknown observer model. In spite of different assumptions, BMS and GDIC agree on the most likely observer model, validating the robustness of our main findings. The two approaches exhibit differences with respect to model ranking, due to the fact that, as a ‘fixed effect’ method, GDIC does not account for group heterogeneity and outliers [31] (see Section 4 in Text S1 for details). Finally, we assessed the impact of each factor on model performance by computing the average change in DIC associated with a given component.

#### Results of model comparison

**Figure 8:**
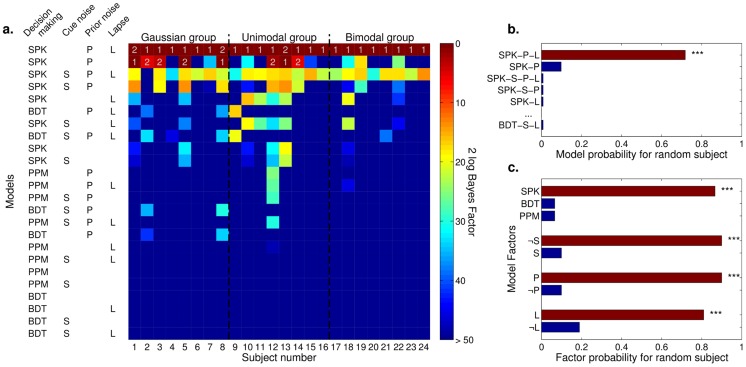
Model comparison between individual models. **a**: Each column represents a subject, divided by test group (all datasets include a Gaussian training session), each row an observer model identified by a model string (see Table 2). Cell color indicates model's evidence, here displayed as the Bayes factor against the best model for that subject (a higher value means a worse performance of a given model with respect to the best model). Models are sorted by their posterior likelihood for a randomly selected subject (see panel b). Numbers above cells specify ranking for most supported models with comparable evidence (difference less than 10 in 2 log Bayes factor [32]). **b**: Probability that a given model generated the data of a randomly chosen subject. Here and in panel c, brown bars represent the most supported models (or model levels within a factor). Asterisks indicate a significant exceedance probability, that is the posterior probability that a given model (or model component) is more likely than any other model (or model component): 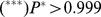. **c**: Probability that a given model level within a factor generated the data of a randomly chosen subject.

Figure 8 shows the results of the BMS method applied to our model set. Figure 8a shows the model evidence for each individual model and subject. For each subject we computed the posterior probability of each observer model using DIC as an approximation of the marginal likelihood (see Methods). We calculated model evidence as the Bayes factor (posterior probability ratio) between the subject's best model and a given model. In the graph we report model evidence in the same scale as DIC, that is as twice the log Bayes factor. A difference of more than 10 in this scale is considered very strong evidence [32]. Results for individual subjects show that model SPK-P-L (stochastic posterior with estimation noise on the prior and lapse) performed consistently better than other models for all conditions. A minority of subjects were also well represented by model SPK-P (same as above, but without the lapse component). All other models performed significantly worse. In particular, note that the richer SPK-S-P-L model was not supported, suggesting that that sensory noise on estimation of cue location was not needed to explain the data. Figure 8b confirms these results by showing the estimated probability of finding a given observer model in the population (assuming that multiple observer models could be present). Model SPK-P-L is significantly more represented (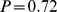; exceedance probability 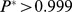), followed by model SPK-P (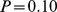). For all other models the probability is essentially the same at 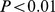. The probability of single model factors reproduced an analogous pattern (Figure 8c). The majority of subjects (more than 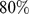 in each case) are likely to use a stochastic decision making (SPK), to have noise in the estimation of the priors (P), and lapse (L). Only a minority (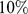) would be described by an observer model with sensory noise in estimation of the cue. The model comparison yielded similar results, although with a more graded difference between models, when looking directly at DIC scores (see Section 4 in Text S1; lower is better).

To assess in another way the relative importance of each model component in determining the performance of a model, we measured the average contribution to DIC of each model level within a factor across all tested models (Figure 4 in Text S1). In agreement with our previous findings, the lowest DIC (better score) in decision making is obtained by observer models containing the SPK factor. BDT increases (i.e. worsens) average DIC scores substantially (difference in DIC, 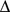
DIC = 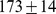; mean 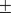 SE across subjects) and PPM has devastating effects on model performance (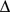
DIC = 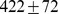), where 10 points of 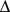
DIC may already be considered a strong evidence towards the model with lower DIC [30]. Regarding the other factors (S, P, L) we found that in general lacking a factor increases DIC (worse model performance; see Section 4 in Text S1 for discussion about factor S). Overall, this analysis confirms the strong impact that an appropriate modelling of variability has on model performance (see Section 4 in Text S1 for details).

We performed a number of analyses on an additional set of observer models to validate the finding that model SPK-P-L provides the best explanation for the data in our model set.

Firstly, in all the observer models described so far the subjects' parameters of the likelihood, 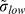 and 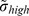, were allowed to vary. Preliminary analysis had suggested that observer models with mismatching likelihoods always outperformed models with true likelihood parameters, 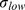 and 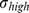. We tested whether this was the case also with our current best model, or if we could assume instead that at least some subjects were using the true parameters. Model SPK-P-L-true performed considerably worse than its counterpart with mismatching likelihood parameters (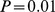 with 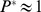 for the other model; 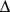
DIC = 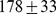), suggesting that mismatching likelihoods are invariably necessary to explain our subjects' data.

We then checked whether the variability of subjects' estimates of the priors may have arisen instead due to the discrete representation of the prior distribution in the experiment (see Figure 1d). We therefore considered a model SPK-D-L in which priors were not noisy, but the model component ‘D’ replaces the continuous representations of the priors with their true discrete representation (a mixture of a hundred narrow Gaussians corresponding to the dots shown on screen). Model SPK-D-L performed worse than model SPK-P-L (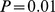 with 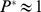 for the other model; 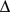
DIC = 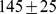) and, more interestingly, also worse than model SPK-L (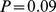 with 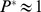 for the other model; 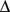
DIC = 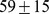). The discrete representation of the prior, therefore, does not provide a better explanation for subjects' behavior.

Lastly, we verified whether our subjects' behavior and apparent variability could be explained by a non-Bayesian iterative model applied to the training datasets. A basic iterative model failed to explain subjects' data (see Section 6 in Text S1 and Discussion).

In conclusion, all analyses identify as the main sources of subjects' suboptimal behavior the combined effect of both noise in estimating the shape of the ‘prior’ distributions and variability in the subsequent decision, plus some occasional lapses.

#### Comparison of alternative models of decision making

Our previous analyses suggest that subjects exhibit variability in decision making that conforms to some nontrivial transformation of the posterior distribution (such as a power function of the posterior, as expressed by model component SPK). We perform a second factorial model comparison that focusses on details of the decision-making process, by including additional model components that describe different transformations of the posterior. We consider in this analysis the following factors (in italic the additions):

1. **Decision making** (4 levels): Bayesian Decision Theory (‘BDT’), stochastic posterior (‘SPK’), posterior probability matching (‘PPM’), *posterior sampling-average* (‘PSA’).
2. **Gaussian approximation of the posterior** (3 levels): no approximation, *mean/variance approximation* (‘MV’) or *Laplace approximation* (‘LA’).
3. **Lapse** (2 levels): absent or present (‘L’).

Our extended model set comprises 18 observer models since some combinations of model factors lead to equivalent observer models. In order to limit the combinatorial explosion of models, in this factorial analysis we do not include model factors S and P that were previously considered, since our main focus here is on decision making (but see below). All new model components are explained in this section and summarized in Table 2.

Firstly, we illustrate an additional level for the decision-making factor. According to model PSA (posterior sampling-average), we assume that the observer chooses a target by taking the average of 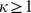 samples drawn from the posterior distribution [33]. This corresponds to an observer with a sample-based posterior that applies a quadratic loss function when choosing the optimal target. For generality, with an interpolation method we allow 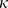 to be a real number (see Methods).

We also introduce a new model factor according to which subjects may use a single Gaussian to approximate the full posterior. The mean/variance model (MV) assumes that subjects approximate the posterior with a Gaussian with matching low-order moments (mean and variance). For observer models that act according to BDT, model MV is equivalent to the assumption of a quadratic loss function during target selection, whose optimal target choice equals the mean of the posterior. Alternatively, a commonly used Gaussian approximation in Bayesian inference is the Laplace approximation (LA) [34]. In this case, the observer approximates the posterior with a single Gaussian centered on the mode of the posterior and whose variance depends on the local curvature at the mode (see Methods). The main difference of the Laplace approximation from other models is that the posterior is usually narrower, since it takes into account only the main peak.

Crucially, the predictions of these additional model components differ only if the posterior distribution is non-Gaussian; these observer models represent different generalizations of how a noisy decision process could affect behavior beyond the Gaussian case. Therefore we include in this analysis only trials in which the theoretical posterior distribution is considerably non-Gaussian (see Methods); this restriction immediately excludes from the analysis the training sessions and the Gaussian group, in which all priors and posteriors are strictly Gaussian.

**Figure 9:**
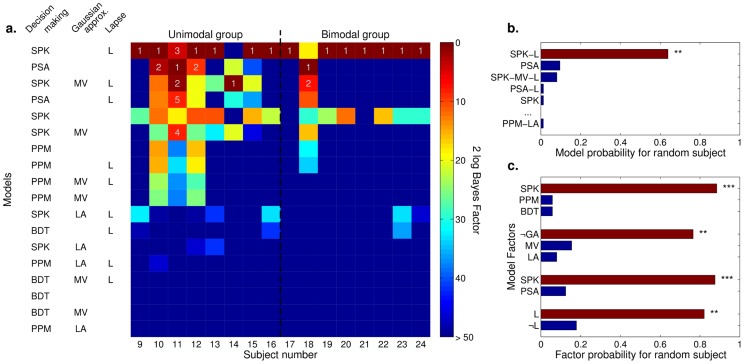
Comparison between alternative models of decision making. We tested a class of alternative models of decision making which differ with respect to predictions for non-Gaussian trials only. **a**: Each column represents a subject, divided by group (either unimodal or bimodal test session), each row an observer model identified by a model string (see Table 2). Cell color indicates model's evidence, here displayed as the Bayes factor against the best model for that subject (a higher value means a worse performance of a given model with respect to the best model). Models are sorted by their posterior likelihood for a randomly selected subject (see panel b). Numbers above cells specify ranking for most supported models with comparable evidence (difference less than 10 in 2 log Bayes factor [32]). **b**: Probability that a given model generated the data of a randomly chosen subject. Here and in panel c, brown bars represent the most supported models (or model levels within a factor). Asterisks indicate a significant exceedance probability, that is the posterior probability that a given model (or model component) is more likely than any other model (or model component): 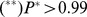, 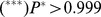. **c**: Probability that a given model level within a factor generated the data of a randomly chosen subject. Label ‘
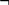
GA’ stands for no Gaussian approximation (full posterior).

Figure 9 shows the results of the BMS method applied to this model set. As before, we consider first the model evidence for each individual model and subject (Figure 9a). Results are slighly different depending on the session (unimodal or bimodal) but in both cases model SPK-L (stochastic posterior with lapse) performs consistently better than other tested models for all conditions. Only a couple of subjects are better described by a different approximation of the posterior (either PSA or SPK-MV-L). These results are summarized in Figure 9b, which shows the estimated probability that a given model would be responsible of generating the data of a randomly chosen subject. We show here results for both groups; a separate analysis of each group did not show qualitative differences. Model SPK-L is significantly more represented (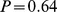; exceedance probability 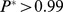), followed by model PSA (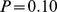) and SPK-MV-L (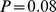). For all other models the probability is essentially the same at 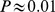. The probability of single model factors reproduces the pattern seen before (Figure 9c). The majority of subjects (more than 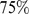 in each case) are likely to use a stochastic decision making (SPK), to use the full posterior (no Gaussian approximations) and lapse (L).

The model comparison performed on group DIC scores (GDIC) obtained mostly similar results although with a more substantial difference between the unimodal group and the bimodal group (Figure 3 in Text S1). In particular, group DIC scores fail to find significant differences between distinct types of approximation of the posterior in the unimodal case. The reason is that for several subjects in the unimodal group differences between models are marginal, and GDIC does not have enough information to disambiguate between these models. Nonetheless, results in the bimodal case are non-ambigous, and overall the SPK-L model emerges again as the best description of subjects' behavior (see Section 4 in Text S1 for details).

As mentioned before, in order to limit model complexity we did not include model factors S and P in the current analysis. We can arguably ignore sensory noise in cue estimation, S, since it was already proven to have marginal effect on subjects' behavior, but this is not the case for noisy estimation of the prior, P. We need, therefore, to verify that our main results about decision making in the case of non-Gaussian posteriors were not affected by the lack of this factor. We compared the four most represented models of the current analysis (Figure 9b) augmented with the P factor: SPK-P-L, PSA-P, SPK-MV-P-L and PSA-P-L. Model SPK-P-L was still the most representative model (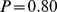, exceedance probability 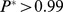), showing that model factor P does not affect our conclusions on alternative models of decision making. We also found that model SPK-P-L obtained more evidence than any other model tested in this section (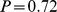, exceedance probability 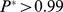), in agreement with the finding of our first factorial model comparison.

Finally, even though the majority of subjects' datasets is better described by the narrow loss function of the task, a few of them support also observer models that subtend a quadratic loss. To explore this diversity, we examined an extended BDT model in which the loss width 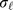 is a free parameter (see Section 3 in Text S1). This model performed slightly better than a BDT model with fixed 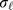, but no better than the equivalent SPK model, so our findings are not affected.

In summary, subjects' variability in our task is compatible with them manipulating the full shape of the posterior corrupted by noise (SPK), and applying a close approximation of the loss function of the task. Our analysis marks as unlikely alternative models of decision making that use instead a quadratic loss or different low-order approximations of the posterior.

### Analysis of best observer model

After establishing model SPK-P-L as the ‘best’ description of the data among the considered observer models, we examined its properties. First of all, we inspected the posterior distribution of the model parameters given the data for each subject. In almost all cases the marginalized posterior distributions were unimodal with a well-defined peak. We therefore summarized each posterior distribution with a point estimate (a robust mean) with minor loss of generality; group averages are listed in Table 3. For the analyses in this section we ignored outlier parameter values that fell more than 3 SDs away from the group mean (this rule excluded at most one value per parameter). In general, we found a reasonable statistical agreement between parameters of different sessions, with some discrepancies in the unimodal test session only. In this section, inferred values are reported as mean 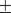 SD across subjects.

**Table 3:** Best observer model's estimated parameters.

Session	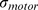	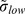	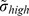	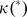	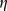	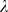
Gaussian training	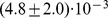	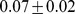	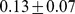	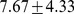	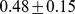	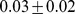
Gaussian test	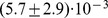	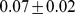	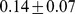	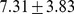	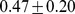	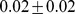
Unimodal test	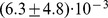	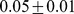	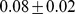	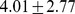	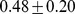	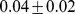
Bimodal test	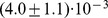	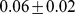	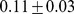	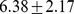	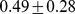	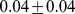
True values	–	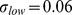	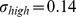	–	–	–

Group-average estimated parameters for the ‘best’ observer model (SPK-P-L), grouped by session (mean 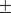 SD across subjects). For each subject, the point estimates of the parameters were computed through a robust mean of the posterior distribution of the parameter given the data. For reference, we also report the true noise values of the cues, 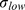 and 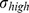. (^*^) We ignored values of 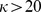.

The motor noise parameter 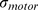 took typical values of 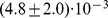 screen units (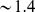 mm), somewhat larger on average than the values found in the sensorimotor estimation experiment, although still in a reasonable range (see Text S3). The inferred amount of motor noise is lower than estimates from previous studies in reaching and pointing (e.g. [10]), but in our task subjects had the possibility to adjust their end-point position.

The internal estimates of cue variability for low-noise and high-noise cues (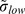 and 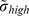) were broadly scattered around the true values (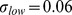 and 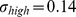 screen units). In general, individual values were in qualitative agreement with the true parameters but showed quantitative discrepancies. Differences were manifest also at the group level, as we found statistically significant disagreement for both low and high-noise cues in the unimodal test session (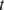-test, 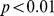) and high-noise cues in the bimodal test session (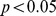). The ratio between the two likelihood parameters, 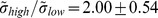, differed significantly from the true ratio, 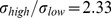 (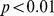).

A few subjects (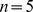) were very precise in their decision-making process, with a power function exponent 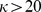. For the majority of subjects, however, 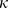 took values between 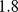 and 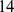 (median 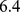), corresponding approximately to an amount of decision noise of 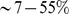 of the variance of the posterior distribution (median 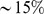). The range of exponents is compatible with values of 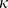 (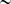 number of samples) previously reported in other experiments, such as a distance-estimation task [33] or ‘intuitive physics’ judgments [35]. In agreement with the results of our previous model comparison, the inferred exponents suggest that subjects' stochastic decision making followed the shape of a considerably narrower version of the posterior distribution (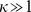) which is not simply a form of posterior-matching (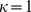).

The Weber's fraction of estimation of the parameters of the priors' density took typical values of 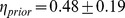, with similar means across conditions. These values denote quite a large amount of noise in estimating (or manipulating) properties of the priors. Nonetheless, such values are in qualitative agreeement with a density/numerosity estimation experiment in which a change of 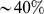 in density or numerosity of a field of random dots was necessary for subjects to note a difference in either property [36]. Although the two tasks are too different to allow a direct quantitative comparison, the thresholds measured in [36] suggest that density/numerosity estimation can indeed be as noisy as we found.

Finally, even though we did not set an informative prior over the parameter, the lapse rate took reasonably low values as expected from a probability of occasional mistakes [28], [37]. We found 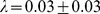, and the inferred lapse rate averaged over training and test session was less than 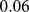 for all but one subject.

**Figure 10:**
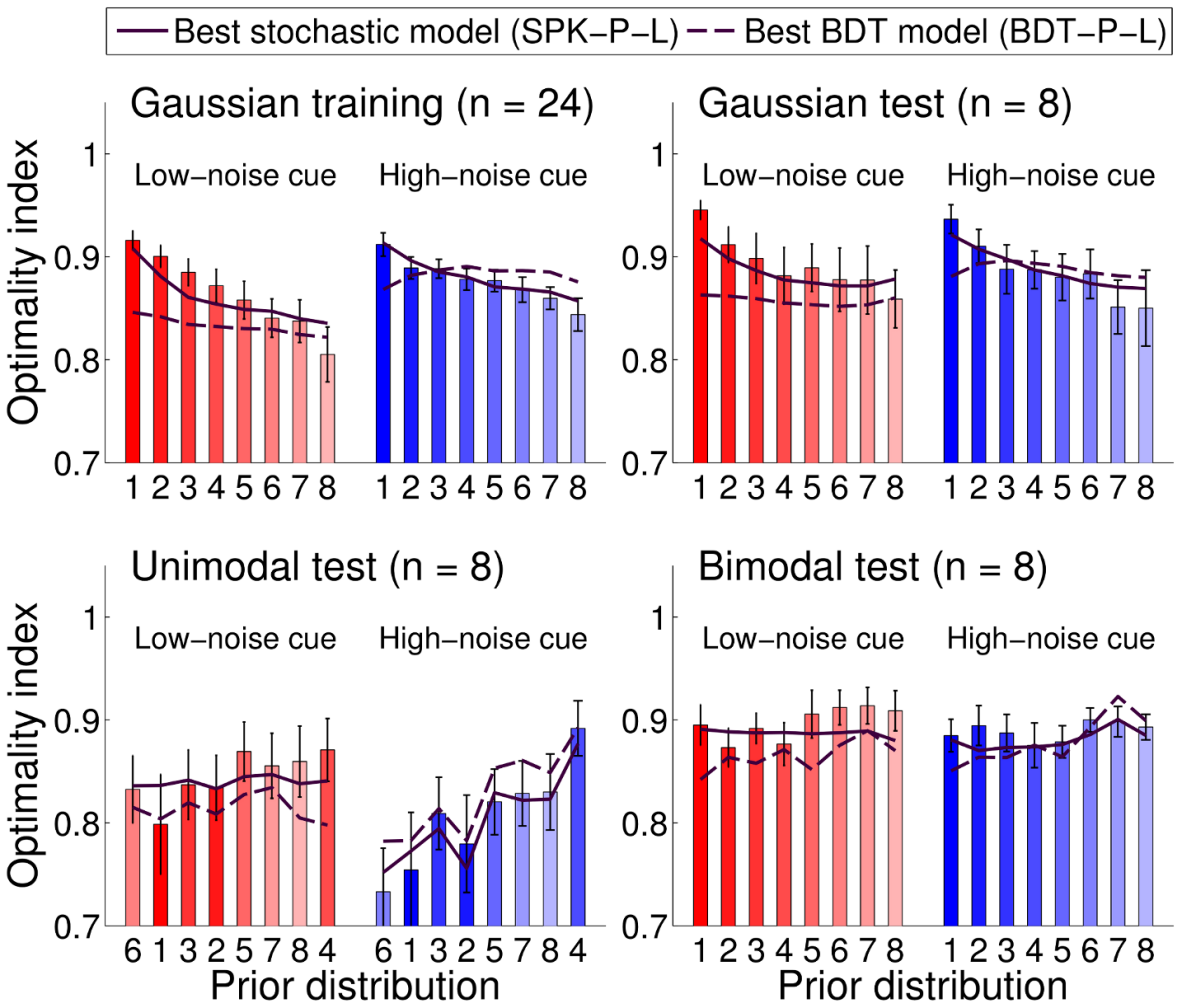
Model ‘postdiction’ of the optimality index. Each bar represents the group-averaged optimality index for a specific session, for each prior (indexed from 1 to 8, see also Figure 2) and cue type, either low-noise cues (red bars) or high-noise cues (blue bars); see also Figure 5. Error bars are SE across subjects. The continuous line represents the ‘postdiction’ of the best suboptimal Bayesian observer model, model SPK-P-L; see ‘Analysis of best observer model’ in the text). For comparison, the dashed line is the ‘postdiction’ of the best suboptimal observer model that follows Bayesian Decision Theory, BDT-P-L.

We examined the best observer model's capability to reproduce our subjects' performance. For each subject and group, we generated 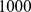 datasets simulating the responses of the SPK-P-L observer model to the experimental trials experienced by the subject. For each simulated dataset, model parameters were sampled from the posterior distribution of the parameters given the data. For each condition (shape of prior and cue type) we then computed the optimality index and averaged it across simulated datasets. The model's ‘postdictions’ are plotted in Figure 10 as continuous lines (SE are omitted for clarity) and appear to be in good agreement with the data. Note that the postdiction is not exactly a fit since (a) the parameters are not optimized specifically to minimize performance error, and (b) the whole posterior distribution of the parameters is used and not just a ‘best’ point estimate. As a comparison, we also plotted in Figure 10 the postdiction for the best BDT observer model, BDT-P-L (dashed line). As the model comparison suggested, standard Bayesian Decision Theory fails to capture subjects' performance.

**Figure 11:**
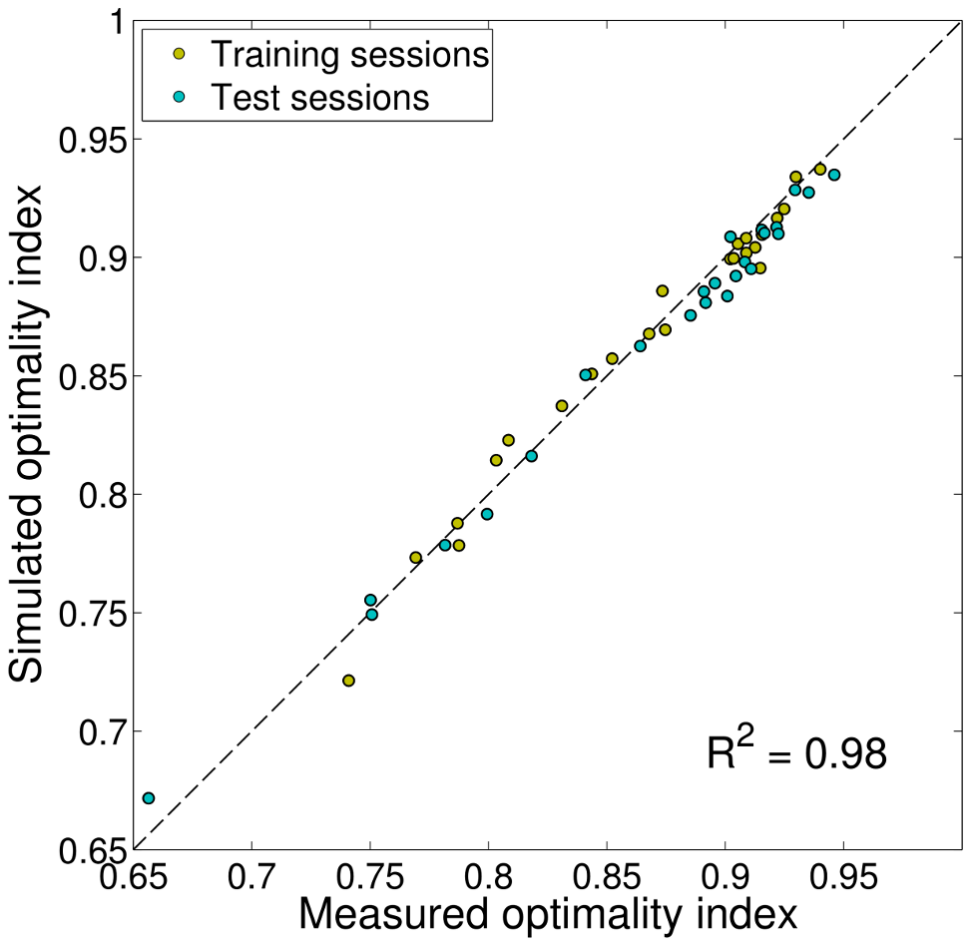
Comparison of measured and simulated performance. Comparison of the mean optimality index computed from the data and the simulated optimality index, according to the ‘postdiction’ of the best observer model (SPK-P-L). Each dot represents a single session for each subject (either training or test). The dashed line corresponds to equality between observed and simulated performance. Model-simulated performance is in good agreement with subjects' performance (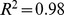).

For each subject and group (training and test) we also plot the mean optimality index of the simulated sessions against the optimality index computed from the data, finding a good correlation (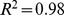; see Figure 11).

**Figure 12:**
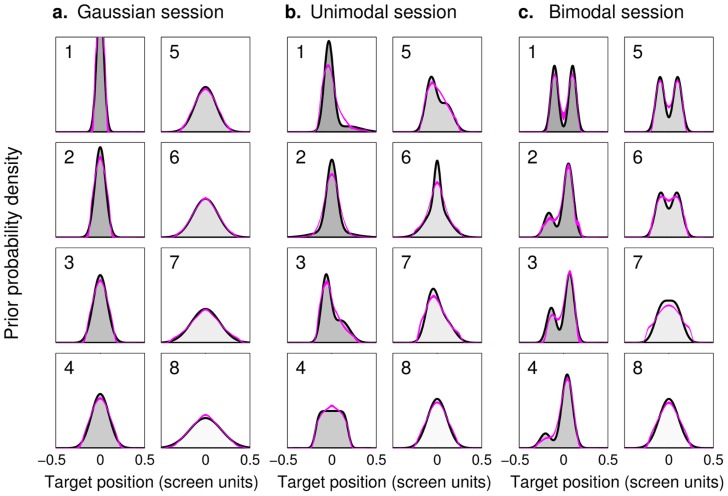
Reconstructed prior distributions. Each panel shows the (unnormalized) probability density for a ‘prior’ distribution of targets, grouped by test session, as per Figure 2. Purple lines are mean reconstructed priors (mean 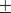 1 s.d.) according to observer model SPK-L. **a: Gaussian session.** Recovered priors in the Gaussian test session are very good approximations of the true priors (comparison between SD of the reconstructed priors and true SD: 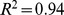). **b: Unimodal session.** Recovered priors in the unimodal test session approximate the true priors (recovered SD: 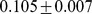, true SD: 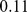 screen units) although with systematic deviations in higher-order moments (comparison between moments of the reconstructed priors and true moments: skewness 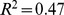; kurtosis 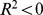). Reconstructed priors are systematically less kurtotic (less peaked, lighter-tailed) than the true priors. **c: Bimodal session.** Recovered priors in the bimodal test session approximate the true priors with only minor systematic deviations (recovered SD: 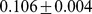, true SD: 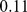 screen units; coefficient of determination between moments of the reconstructed priors and true moments: skewness 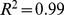; kurtosis 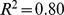).

Lastly, to gain an insight on subjects' systematic response biases, we used our framework in order to nonparametrically reconstruct what the subjects' priors in the various conditions would look like [2], [3], [8], [9] (see Methods). Due to limited data per condition and computational constraints, we recovered the subjects' priors at the group level and for model SPK-L, without additional noise on the priors (P). The reconstructed average priors for distinct test sessions are shown in Figure 12. Reconstructed priors display a very good match with the true priors for the Gaussian session and show minor deviations in the other sessions. The ability of the model to reconstruct the priors – modulo residual idiosyncrasies – is indicative of the goodness of the observer model in capturing subjects' sources of suboptimality.

## Discussion

We have explored human performance in probabilistic inference (a target estimation task) for different classes of prior distributions and different levels of reliability of the cues. Crucially, in our setup subjects were required to perform Bayesian computations with explicitly provided probabilistic information, thereby removing the need either for statistical learning or for memory and recall of a prior distribution. We found that subjects performed suboptimally in our paradigm but that their relative degree of suboptimality was similar across different priors and different cue noise. Based on a generative model of the task we built a set of suboptimal Bayesian observer models. Different methods of model comparison among this large class of models converged in identifying a most likely observer model that deviates from the optimal Bayesian observer in the following points: (a) a mismatching representation of the likelihood parameters, (b) a noisy estimation of the parameters of the prior, (c) a few occasional lapses, and (d) a stochastic representation of the posterior (such that the target choice distribution is approximated by a power function of the posterior).

### Human performance in probabilistic inference

Subjects integrated probabilistic information from both prior and cue in our task, but rarely exhibited the signature of full ‘synergistic integration’, i.e. a performance above that which could be obtained by using either the prior or the cue alone (see Figure 5). However, unlike most studies of Bayesian learning, on each trial in our study subjects were presented with a new prior. A previous study on movement planning with probabilistic information (and fewer conditions) similarly found that subjects violated conditions of optimality [23].

More interestingly, in our data the relative degree of suboptimality did not show substantial differences across distinct classes of priors and noise levels of the cue (low-noise and high-noise). This finding suggests that human efficacy at probabilistic inference is only mildly affected by complexity of the prior per se, at least for the distributions we have used. Conversely, the process of learning priors is considerably affected by the class of the distribution: for instance, learning a bimodal prior (when it is learnt at all) can require thousands of trials [9], whereas mean and variance of a single Gaussian can be acquired reliably within a few hundred trials [11].

Within the same session, subjects' relative performance was influenced by the specific shape of the prior. In particular, for Gaussian priors we found a systematic effect of the variance – subjects performed worse with wider priors, more than what would be expected by taking into account the objective decrease in available information. Interestingly, neither noise in estimation of the prior width (factor P) nor occasional lapses that follow the shape of the prior itself (factor L) are sufficient to explain this effect. Model postdictions of model BDT-P-L show large systematic deviations from subjects' performance in the Gaussian sessions, whereas the best model with decision noise, SPK-P-L, is able to capture subjects' behavior; see top left and top right panels in Figure 10. Moreover, the Gaussian priors recovered under model SPK-L match extremely well the true priors, furthering the role of the stochastic posterior in fully explaining subjects' performance with Gaussians. The crucial aspect of model SPK may be that decision noise is proportional to the width of the posterior, and not merely of the prior.

In the unimodal test session, subjects' performance was positively correlated with the width of the main peak of the distribution. That is, non-Gaussian, narrow-peaked priors (such as priors 1 and 6 in Figure 12b) induced worse performance than broad and smooth distributions (e.g. priors 4 and 8). Subjects tended to ‘mistrust’ the prior, especially in the high-noise condition, giving excess weight to the cue (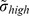 is significantly lower than it should be; see Table 3), which can be also interpreted as an overestimation of the width of the prior. In agreement with this description, the reconstructed priors in Figure 12b show a general tendency to overestimate the width of the narrower peaks, as we found in a previous study of interval timing [8]. This behavior is compatible with a well-known human tendency of underestimating (or, alternatively, underweighting) the probability of occurrence of highly probable results and overestimating (overweighting) the frequency of rare events (see [27], [38], [39]). Similar biases in estimating and manipulating prior distributions may be explained with an hyperprior that favors more entropic and, therefore, smoother priors in order to avoid ‘overfitting’ to the environment [40].

### Modelling suboptimality

In building our observer models we made several assumptions. For all models we assumed that the prior adopted by observers in Eq. 2 corresponded to a continuous approximation of the probability density function displayed on screen, or a noisy estimate thereof. We verified that using the original discrete representation does not improve model performance. Clearly, subjects may have been affected by the discretization of the prior in other ways, but we assumed that such errors could be absorbed by other model components. We also assumed subjects quickly acquired a correct internal model of the probabilistic structure of the task, through practice and feedback, although quantitative details (i.e. model parameters) could be mismatched with respect to the true parameters. Formally, our observer models were not ‘actor’ models in the sense that they did not take into account the motor error in the computation of the expected loss. However, this was with negligible loss of generality since the motor term has no influence on the inference of the optimal target for single Gaussians priors, and yields empirically negligible impact for other priors for small values of the motor error 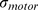 (as those measured in our task; see Text S3).

Suboptimality was introduced into our observer models in three main ways: (a) miscalibration of the parameters of the likelihood; (b) models of approximate inference; and (c) additional stochasticity, either on the sensory inputs or in the decision-making process itself. Motor noise was another source of suboptimality, but its contribution was comparably low.

Miscalibration of the parameters of the likelihood means that the subjective estimates of the reliability of the cues (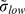 and 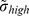) could differ from the true values (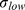 and 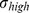). In fact, we found slight to moderate discrepancies, which became substantial in some conditions. Previous studies have investigated whether subjects have (or develop) a correct internal estimate of relevant noise parameters (i.e. the likelihood) which may correspond to their own sensory or motor variability plus some externally injected noise. In several cases subjects were found to have a miscalibrated model of their own variability which led to suboptimal behavior [33], [41]–[43], although there are cases in which subjects were able to develop correct estimates of such parameters [10], [44], [45].

More generally, it could be that subjects were not only using incorrect parameters for the task, but built a wrong internal model or were employing approximations in the inference process. For our task, which has a relatively simple one-dimensional structure, we did not find evidence that subjects were using low-order approximations of the posterior distribution. Also, the capability of our models to recover the subjects' priors in good agreement with the true priors suggest that subjects' internal model of the task was not too discrepant from the true one.

Crucial element in all our models was the inclusion of extra sources of variability, in particular in decision making. Whereas most forms of added noise have a clear interpretation, such as sensory noise in the estimation of the cue location, or in estimating the parameters of the prior, the so-called ‘stochastic posterior’ deserves an extended explanation.

### Understanding the stochastic posterior

We introduced the stochastic posterior model of decision making, SPK, with two intuitive interpretations, that is a noisy posterior or a sample-based approximation (see Figure 7 and Text S2), but clearly any process that produces a variability in the target choice distribution that approximates a power function of the posterior is a candidate explanation. The stochastic posterior captures the main trait of decision noise, that is a variability that depends on the shape of the posterior [33], as opposed to other forms of noise that do not depend on the decision process. Outstanding open questions are therefore which kind of process could be behind the observed noise in decision making, and during which stage it arises, e.g. whether it is due to inference or to action selection [46].

A seemingly promising candidate for the source of noise in the inference is neuronal variability in the nervous system [47]. Although the noisy representation of the posterior distribution in Figure 7b through a population of units may be a simplistic cartoon, the posterior could be encoded in subtler ways (see for instance [48]). However, neuronal noise itself may not be enough to explain the amount of observed variability (see Text S2). An extension of this hypothesis is that the noise may emerge since suboptimal computations magnify the underlying variability [49].

Conversely, another scenario is represented by the sampling hypothesis, an approximate algorithm for probabilistic inference which could be implemented at the neural level [19]. Our analysis ruled out an observer whose decision-making process consists in taking the average of 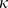 samples from the posterior – operation that implicitly assumes a quadratic loss function – showing that averaging samples from the posterior is not a generally valid approach, although differences can be small for unimodal distributions. More generally, the sampling method should always take into account the loss function of the task, which in our case is closer to a delta function (a MAP solution) rather than to a quadratic loss. Our results are compatible with a proper sampling approach, in which an empirical distribution is built out of a small number of samples from the posterior, and then the expected loss is computed from the sampled distribution [19].

As a more cognitive explanation, decision variability may have arisen because subjects adopted a probabilistic instead of deterministic strategy in action selection as a form of exploratory behavior. In reinforcement learning this is analogous to the implementation of a probabilistic policy as opposed to a deterministic policy, with a ‘temperature’ parameter that governs the amount of variability [50]. Search strategies have been hypothesized to lie behind suboptimal behaviors that appear random, such as probability matching [51]. While generic exploratory behavior is compatible with our findings, our analysis rejected a simple posterior-matching strategy [25], [26].

All of these interpretations assume that there is some noise in the decision process itself. However, the noise could emerge from other sources, without the necessity of introducing deviations from standard BDT. For instance, variability in the experiment could arise from lack of stationarity: dependencies between trials, fluctuations of subjects' parameters or time-varying strategies would appear as additional noise in a stationary model [52]. We explored the possibility of nonstationary behavior without finding evidence for strong effects of nonstationarity (see Section 6 in Text S1). In particular, an iterative (trial-dependent) non-Bayesian model failed to model the data in the training dataset better than the stochastic posterior model. Clearly, this does not exclude that different, possibly Bayesian, iterative models could explain the data better, but our task design with multiple alternating conditions and partial feedback should mitigate the effect of dependencies between trials, since each trial typically displays a different condition from the immediately preceding ones.

In summary, we show that a decision strategy that implements a ‘stochastic posterior’ that introduces variability in the computation of the expected loss has several theoretical and empirical advantages when modelling subjects' performance, demonstrating improvement over previous models that implemented variability only through a ‘posterior-matching’ approach or that implicitly assume a quadratic loss function (sampling-average methods).

## Methods

### Ethics statement

The Cambridge Psychology Research Ethics Committee approved the experimental procedures and all subjects gave informed consent.

### Participants

Twenty-four subjects (10 male and 14 female; age range 18–33 years) participated in the study. All participants were naïve to the purpose of the study. All participants were right-handed according to the Edinburgh handedness inventory [53], with normal or corrected-to-normal vision and reported no neurological disorder. Participants were compensated for their time.

### Behavioral task

Subjects were required to reach to an unknown target given probabilistic information about its position. Information consisted of a visual representation of the a priori probability distribution of targets for that trial and a noisy cue about the actual target position.

Subjects held the handle of a robotic manipulandum (vBOT, [54]). The visual scene from a CRT monitor (Dell UltraScan P1110, 21-inch, 100 Hz refresh rate) was projected into the plane of the hand via a mirror (Figure 1a) that prevented the subjects from seeing their hand. The workspace origin, coordinates 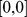, was 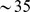 cm from the torso of the subjects, with positive axes towards the right (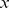 axis) and away from the subject (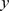 axis). The workspace showed a home position (1.5 cm radius circle) at 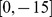 cm and a cursor (1.25 cm radius circle) that tracked the hand position.

On each trial 100 potential targets (0.1 cm radius dots) were shown around the target line at positions 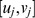, for 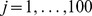, where the 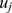 formed a fixed discrete representation of the trial-dependent ‘prior’ distribution 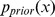, obtained through a regular sample of the cdf (see Figure 1d), and the 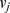 were small random offsets used to facilitate visualization (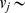 Uniform(−0.3, 0.3) cm). The true target was chosen by picking one of the potential targets at random with uniform probability. A cue (0.25 cm radius circle) was shown at position 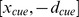. The horizontal position 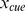 provided a noisy estimate of the target position, 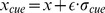, with 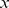 the true (horizontal) position of the target, 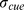 the cue variability and 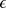 a normal random variable with zero mean and unit variance. The distance of the cue from the target line, 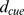, was linearly related to the cue variability: cues distant from the target line were noisier than cues close to it. In our setup, the noise level 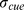 could only either be low for ‘short-distance’ cues, 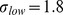 cm (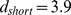 cm), or high for ‘long-distance’ cues, 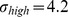 cm (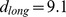 cm). Both the prior distribution and cue remained on the screen for the duration of a trial.

After a ‘go’ beep, subjects were required to move the handle towards the target line, choosing an endpoint position such that the true target would be within the cursor radius. The manipulandum generated a spring force along the depth axis (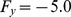 N/cm) for cursor positions past the target line, preventing subjects from overshooting. The horizontal endpoint position of the movement (velocity of the cursor less than 0.5 cm/s), after contact with the target line, was recorded as the subject’s response 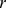 for that trial.

At the end of each trial, subjects received visual feedback on whether their cursor encircled (a ‘success’) or missed the true target (partial feedback). On full feedback trials, the position of the true target was also shown (0.25 cm radius yellow circle). Feedback remained on screen for 1 s. Potential targets, cues and feedback then disappeared. A new trial started 500 ms after the subject had returned to the home position.

For simplicity, all distances in the experiment are reported in terms of standardized screen units (window width of 1.0), with 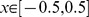 and 0.01 screen units corresponding to 3 mm. In screen units, the cursor radius is 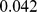 and the SD of noise for short and long distance cues is respectively 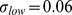 and 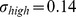.

### Experimental sessions

Subjects performed one practice block in which they were familiarized with the task (64 trials). The main experiment consisted of a training session with Gaussian priors (576 trials) followed by a test session with group-dependent priors (576–640 trials). Sessions were divided in four runs. Subjects could take short breaks between runs and there was a mandatory 15 minutes break between the training and test sessions.

Each session presented eight different types of priors and two cue noise levels (corresponding to either ‘short’ or ‘long’ cues), for a total of 16 different conditions (36–40 trials per condition). Trials from different conditions were presented in random order. Depending on the session and group, priors belonged to one of the following classes (see Figure 2):

#### Gaussian priors

Eight Gaussian distributions with evenly spread SDs between 0.04 and 0.18 i.e. 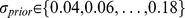 screen units.

#### Unimodal priors

Eight unimodal priors with fixed SD 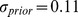 and variable skewness and kurtosis. With the exception of platykurtic prior 4, which is a mixture of 11 Gaussians, and prior 8, which is a single Gaussian, all other priors were realized as mixtures of two Gaussians that locally maximize differential entropy for given values of the first four central moments. In the maximization we included a constraint on the SDs of the individual components so to prevent degenerate solutions (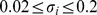 screen units, for 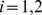). Skewness and excess kurtosis were chosen to represent various shapes of unimodal distributions, within the strict bounds that exist between skewness and kurtosis of a unimodal distribution [55]. The values of (skewness, kurtosis) for the eight distributions, in order of increasing differential entropy: 1: 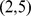; 2: 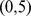; 3: 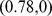; 4: 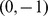; 5: 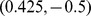; 6: 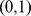; 7: 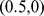; 8: 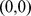.

#### Bimodal priors

Eight (mostly) bimodal priors with fixed SD 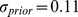 and variable separation and relative weight. The priors were realized as mixtures of two Gaussians with equal variance: 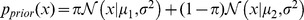. Separation was computed as 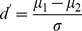, and relative weight was defined as 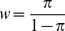. The values of (separation, relative weight) for the eight distributions, in order of increasing differential entropy: 1: 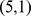; 2: 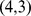; 3: 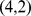; 4: 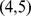; 5: 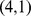; 6: 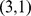; 7: 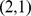; 8: 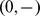 (the last distribution is a single Gaussian).

For all priors, the mean 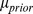 was drawn from a uniform distribution whose bounds were chosen such that the extremes of the discrete representation would fall within the active screen window (the actual screen size was larger than the active window). Also, asymmetric priors had 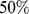 probability of being flipped horizontally about the mean.

### Data analysis

#### Analysis of behavioral data

Data analysis was conducted in MATLAB 2010b (Mathworks, U.S.A.). To avoid edge artifacts in subjects' response, we discarded trials in which the cue position, 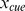, was outside the range of the discretized prior distribution (2691 out of 28672 trials: 9.4%). We included these trials in the experimental session in order to preserve the probabilistic relationships between variables of the task.

For each trial, we recorded the response location 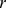 and the reaction time (RT) was defined as the interval between the ‘go’ beep and the start of the subject's movement. For each subject and session we computed a nonlinear kernel regression estimate of the average RT as a function of the SD of the posterior distribution, 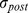. We only considered a range of 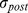 for which all subjects had a significant density of data points. Results did not change qualitatively for other measures of spread of the posterior, such as the exponential entropy [24].

All subjects' datasets are available online in Dataset S1.

#### Optimality index and success probability

We calculated the optimality index for each trial as the success probability for response 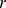, 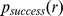, divided by the maximal success probability 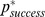, which we used to quantify performance of a subject (or an observer model). The optimality index of our subjects in the task is plotted in Figure 5 and success probabilities are shown in Figure 1 in Text S1.

The success probability 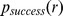 in a given trial represents the probability of locating the correct target according to the generative model of the task (independent of the actual position of the target). For a trial with cue position 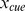, cue noise variance 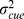, and prior distribution 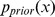, the success probability is defined as: 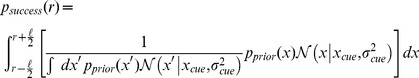
(12)where the integrand is the posterior distribution according to the continuous generative model of the task and 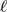 is the diameter of the cursor. Solving the integral in Eq. 12 for a generic mixture-of-Gaussians prior, 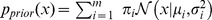, we obtain:
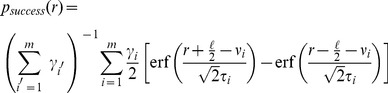
(13)where the symbols 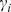, 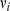 and 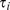 have been defined in Eq. 5. The maximal success probability is simply computed as 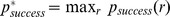.

Note that a metric based on the theoretical success probability is more appropriate than the observed fraction of successes for a given sample of trials, as the latter introduces additional error due to mere chance (the observed fraction of successes fluctuates around the true success probability with binomial statistics, and the error can be substantial for small sample size).

The priors for the Gaussian, unimodal and bimodal sessions were chosen such that the average maximal success probability of each class was about the same (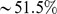) making the task challenging and of equal difficulty across the task.

#### Computing the optimal target

According to Bayesian Decision Theory (BDT), the key quantity an observer needs to compute in order to make a decision is the (subjectively) expected loss for a given action. In our task, the action corresponds to a choice of a cursor position 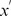, and the expected loss takes the form: 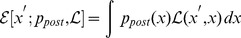
(14)where 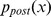 is the subject's posterior distribution of target position, described by Eq. 2, and the loss associated with choosing position 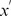 when the target location is 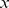 is represented by loss function 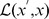.

Our task has a clear ‘hit or miss’ structure that is represented by the square well function: 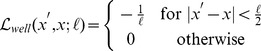
(15)where 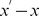 is the distance of the chosen response from the target, and 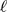 is the size of the allowed window for locating the target (in the experiment, the cursor diameter). The square well loss allows for an analytical expression of the expected loss, but the optimal target still needs to be computed numerically. Therefore we make a smooth approximation to the square well loss represented by the inverted Gaussian loss:
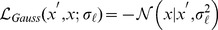
(16)where the parameter 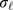 governs the scale of smoothed detection window. The Gaussian loss approximates extremely well the predictions of the square well loss in our task, to the point that performance under the two forms of loss is empirically indistinguishable (see Section 3 in Text S1). However, computationally the Gaussian loss is preferrable as it allows much faster calculations of optimal behavior.

For the decision process, BDT assumes that observers choose the ‘optimal’ target position 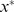 that minimizes the expected loss: 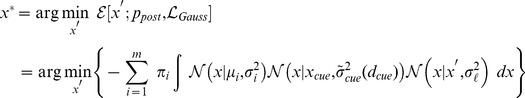
(17)where we have used Eqs. 2, 14 and 16. With some algebraic manipulations, Eq. 17 can be reformulated as Eq. 4. Given the form of the expected loss, the solution of Eq. 4 is equivalent to finding the maximum (mode) of a Gaussian mixture model. In general no analytical solution is known for more than one model component (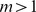), so we implemented a fast and accurate numerical solution adapting the algorithm in [56].

#### Computing the response probability

The probability of observing response 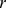 in a trial, 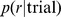 (e.g., Eq. 6) is the key quantity for our probabilistic modelling of the task. For basic observer models, 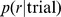 is obtained as the convolution between a Gaussian distribution (motor noise) and a target choice distribution in closed form (e.g. a power function of a mixture of Gaussians), such as in Eqs. 3, 7 and 11. Response probabilities are integrated over latent variables of model factor S (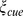; see Eq. 8) and of model factor P (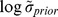 and 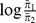; see Eqs. 9 and 10). Integrations were performed analytically when possible or otherwise numerically (trapz in MATLAB or Gauss-Hermite quadrature method for non-analytical Gaussian integrals [57]). For instance, the observed response probability for model factor S takes the shape: 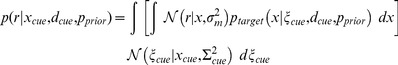
(18)where we are integrating over the hidden variables 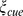 and 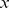. The target choice distribution 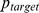 depends on the decision-making model component (see e.g. Eqs. 3 and 7). Without loss of generality, we assumed that the observers are not aware of their internal variability. Predictions of model S do not change whether we assume that the observer is aware of his or her measurement error 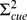 or not; differences amount just to redefinitions of 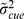.

For a Gaussian prior with mean 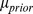 and variance 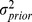, the response probability has the following closed form solution: 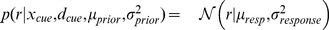
(19)with
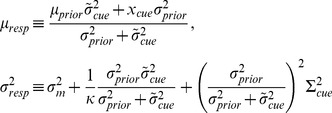
(20)where 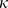 is the noise parameter of the stochastic posterior in model component SPK (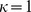 for PPM; 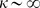 for BDT) and 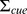 is the sensory noise in estimation of the cue position in model S (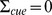 for observer models without cue-estimation noise). For observer models P with noise on the prior, Eq. 19 was numerically integrated over different values of the internal measurement (here corresponding to log 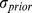) with a Gauss-Hermite quadrature method [57].

For non-Gaussian priors there is no closed form solution similar to Eq. 19 and the calculation of the response probability, depending on active model components, may require up to three nested numerical integrations. Therefore, for computational tractability, we occasionally restricted our analysis to a subset of observer models, as indicated in the main text.

For model class PSA (posterior sampling average), the target choice distribution is the probability distribution of the average of 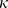 samples drawn from the posterior distribution. For a posterior that is a mixture of Gaussians and integer 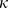, it is possible to obtain an explicit expression whose number of terms grows exponentially in 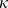. Fortunately, this did not constitute a problem as observer models favored small values of 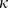 (also, a Gaussian approximation applies for large values of 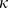 due to the central limit theorem). Values of the distribution for non-integer 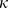 were found by linear interpolation between adjacent integer values. For model class LA (Laplace approximation) we found the mode of the posterior numerically [56] and analytically evaluated the second derivative of the log posterior at the mode. The mean of the approximate Gaussian posterior is set to the mode and the variance to minus the inverse of the second derivative [34].

For all models, when using the model-dependent response probability, 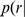
trial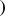, in the model comparison, we added a small regularization term: 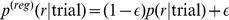
(21)with 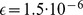 (the value of the pdf of a normal distribution at 5 SDs from the mean). This change in probability is empirically negligible, but from the point of view of model comparison the regularization term introduces a lower bound 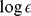 on the log probability of a single trial, preventing single outliers from having unlimited weight on the log likelihood of a model, increasing therefore the robustness of the inference.

#### Sampling and model comparison

For each observer model and each subject's dataset (comprised of training and test session) we calculated the posterior distribution of the model parameters given the data, Pr(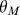 | data, model) 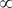 Pr(data| 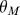, model) Pr(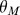 | model), where we assumed a factorized prior over parameters, Pr(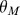 | model)  = 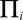 Pr(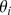 | model). Having obtained independent measures of typical sensorimotor noise parameters of the subjects in a sensorimotor estimation experiment, we took informative log-normal priors on parameters 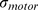 and 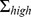 (when present), with log-scale respectively 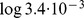 and 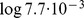 screen units and shape parameters 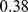 and 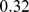 (see Text S3; results did not depend crucially on the shape of the priors). For the other parameters we took a noninformative uniform prior 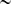 Uniform[0, 1] (dimensionful parameters were measured in normalized screen units), with the exception of the 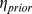 and 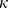 parameters. The 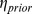 parameter that regulates the noise in the prior could occasionally be quite large (see main text) so we adopted a broader range 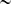 Uniform[0, 4] to avoid edge effects. A priori, the 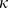 parameter that governs noise in decision making could take any positive nonzero value (with higher probability mass on lower values), so we assumed a prior 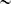 Uniform[0, 1] on 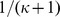, which is equivalent to a prior 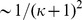, for 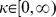. Formally, a value of 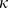 less than one represents a performance more variable than posterior-matching (for 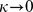 the posterior distribution tends to a uniform distribution). Results of the model comparison were essentially identical whether we allowed 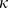 to be less than one or not. We took a prior 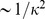 on the positive real line since it is integrable; an improper prior such as a noninformative prior 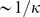 is not recommendable in a model comparison between models with non-common parameters due to the ‘marginalization paradox’ [58].

The posterior distribution of the parameters is proportional to the data likelihood, which was computed in logarithmic form as: 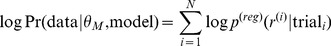
(22)where 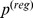 is the regularized probability of response given by Eq. 21, and trial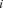 represents all the relevant variables of the 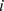-th trial. Eq. 22 assumes that the trials are independent and that subjects' parameters are fixed throughout each session (stationarity). The possibility of dependencies between trials and nonstationarity in the data is explored in Section 6 of Text S1.

A convenient way to compute a probability distribution whose unnormalized pdf is known (Eq. 22) is by using a Markov Chain Monte Carlo method (e.g. slice sampling [29]). For each dataset and model, we ran three parallel chains with different starting points (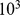 to 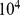 burn-in samples, 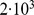 to 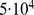 saved samples per chain, depending on model complexity) obtaining a total of 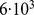 to 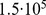 sampled parameter vectors. Marginal pdfs of sampled chains were visually checked for convergence. We also searched for the global minimum of the (minus log) marginal likelihood by running a minimization algorithm (fminsearch in MATLAB) from several starting points (30 to 100 random locations). With this information we verified that, as far as we could tell, the chains were not stuck in a local minimum. Finally, we computed Gelman and Rubin's potential scale reduction statistic 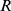 for all parameters [59]. Large values of 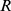 indicate convergence problems whereas values close to 1 suggest convergence. Longer chains were run when suspicion of a convergence problem arose from any of these methods. In the end average 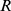 (across parameters, participants and models) was 1.003 and almost all values were 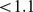 suggesting good convergence.

Given the parameter samples, we computed the DIC score (deviance information criterion) [30] for each dataset and model. The DIC score is a metric that combines a goodness of fit term and a penality for model complexity, similarly to other metrics adopted in model comparison, such as Akaike Information Criterion (AIC) and Bayesian Information Criterion (BIC), with the advantage that DIC takes into account an estimate of the effective complexity of the model and it is particularly easy to compute given a MCMC output. DIC scores are computed as: 
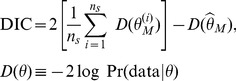
(23)where 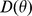 is the *deviance* given parameter vector 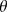, the 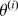 are MCMC parameter samples and 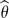 is a ‘good’ parameter estimate for the model (e.g. the mean, median or another measure of central tendency of the sampled parameters). As a robust estimate of 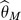 we computed a trimmed mean (discarding 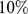 from each side, which eliminated outlier parameter values). DIC scores are meaningful only in a comparison, so we only report DIC scores differences between models (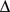
DIC). Although a difference of 3-7 points is already suggested to be significant [30], we follow a conservative stance, for which the difference in DIC scores needs to be 10 or more to be considered significant [33]. In Section 4 of Text S1 we report a set of model comparisons evaluated in terms of group DIC (GDIC). The assumption of GDIC is that all participants' datasets have been generated by the same observer model, and all subjects contribute equally to the evidence of each model.

In the main text, instead, we compared models according to a hierarchical Bayesian model selection method (BMS) [31] that treats both subjects and models as random factors, that is, multiple observer models may be present in the population. BMS uses an iterative algorithm based on variational inference to compute model evidence from individual subjects' marginal likelihoods (or approximations thereof, such as DIC, with the marginal likelihood being 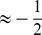 DIC). BMS is particularly appealing because it naturally deals with group heterogeneity and outliers. Moreover, the output of the algorithm has an immediate interpretation as the probability that a given model is responsible for generating the data of a randomly chosen subject. BMS also allows to easily compute the cumulative evidence for groups of models and we used this feature to compare distinct levels within factors [31]. As a Bayesian metric of significance we report the exceedance probability 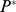 of a model (or model level within a factor) being more likely than any other model (or level). We consider values of 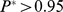 to be significant. The BMS algorithm is typically initialized with a symmetric Dirichlet distribution that represents a prior over model probabilities with no preference for any specific model [31]. Since we are comparing a large number of models generated by the factorial method, we chose for the concentration parameter of the Dirichlet distribution a value 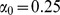 that corresponds to a weak prior belief that only a few observer models are actually present in the population (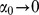 would correspond to the prior belief that only one model is true, similarly to GDIC, and 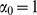 that any number of models are true). Results are qualitatively independent of the specific choice of 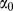 for a large range of values.

When looking at alternative models of decision making in our second factorial model comparison, we excluded from the analysis ‘uninteresting’ trials in which the theoretical posterior distribution (Eq. 2 with the true values of 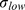 and 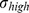) was too close in shape to a Gaussian; since predictions of these models are identical for Gaussian posteriors, Gaussian trials constitute only a confound for the model comparison. A posterior distribution was considered ‘too close’ to a Gaussian if the Kullback-Leibler divergence between a Gaussian approximation with matching low-order moments and the full posterior was less than a threshold value of 0.02 nats (results were qualitatively independent of the chosen threshold). In general, this preprocessing step removed about 45–60% of trials from unimodal and bimodal sessions (clearly, Gaussian sessions were automatically excluded).

#### Nonparametric reconstruction of the priors

We reconstructed the group priors as a means to visualize the subjects’ common systematic biases under a specific observer model (SPK-L). Each group prior 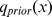 was ‘nonparametrically’ represented by a mixture of Gaussians with a large number of components (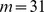). The components' means were equally spaced on a grid that spanned the range of the discrete representation of the prior; SDs were equal to the grid spacing. The mixing weights 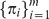 were free to vary to define the shape of the prior (we enforced symmetric values on symmetric distributions, and the sum of the weigths to be one). The representation of the prior as a mixture of Gaussians allowed us to cover a large class of smooth distributions using the same framework as the rest of our study.

For this analysis we fixed subjects' parameters to the values inferred in our main model comparison for model SPK-L (i.e. to the robust means of the posterior of the parameters). For each prior in each group (Gaussian, unimodal and bimodal test sessions), we simultaneously inferred the shape of the nonparametric prior that explained each subject's dataset, assuming the same distribution 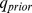 for all subjects. Specifically, we sampled from the posterior distribution of the parameters of the group priors, Pr(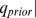 data), with a flat prior over log values of the mixing weights 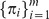. We ran 5 parallel chains with a burn-in of 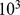 samples and 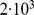 samples per chain, for a total of 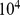 sampled vectors of mixing weights (see previous section for details on sampling). Each sampled vector of mixing weights corresponds to a prior 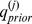, for 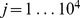. Purple lines in Figure 12 show the mean (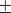 1 SD) of the sampled priors, that is the average reconstructed priors (smoothed with a small Gaussian kernel for visualization purposes). For each sampled prior we also computed the first four central moments (mean, variance, skewness and kurtosis) and calculated the posterior average of the moments (see Figure 12).

#### Statistical analyses

All regressions in our analyses used a robust procedure, computed using Tukey's ‘bisquare’ weighting function (robustfit in MATLAB). Robust means were computed as trimmed means, discarding 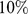 of values from each side of the sample. Statistical differences were assessed using repeated-measures ANOVA (rm-ANOVA) with Greenhouse-Geisser correction of the degrees of freedom in order to account for deviations from sphericity [60]. A logit transform was applied to the optimality index measure before performing rm-ANOVA, in order to improve normality of the data (results were qualitatively similar for non-transformed data). Nonlinear kernel regression estimates to visualize mean data (Figure 3 and 6) were computed with a Nadaraya-Watson estimator with rule-of-thumb bandwidth [61]. For all analyses the criterion for statistical significance was 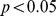.

## Supporting Information

Dataset S1
**Subject's datasets.** Subjects' datasets for the main experiment (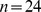, training and test sessions) and for the sensorimotor estimation experiment (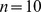), with relevant metadata, in a single MATLAB data file.(ZIP)

Text S1 **Additional analyses and observer models.** This supporting text includes sections on: Translational invariance of subjects' behavior; Success probability; Inverted Gaussian loss function; Model comparison with DIC; Model comparison for different shared parameters between sessions; Nonstationary analysis.
(PDF)

Text S2 **Noisy probabilistic inference.** Description of the models of stochastic probabilistic inference (‘noisy posterior’ and ‘sample-based posterior’) and discussion about unstructured noise in the prior.
(PDF)

Text S3 **Sensorimotor estimation experiment.** Methods and results of the additional experiment to estimate the range of subjects' sensorimotor parameters.
(PDF)
