# Supplementary material for: On the Origins of Suboptimality in Human Probabilistic Inference
Source: PLoS Comput Biol. 2014 Jun 19;10(6):e1003661. doi: 10.1371/journal.pcbi.1003661 (PMC4063671; doi:10.1371/journal.pcbi.1003661)
Supplement: Text S1 — Additional analyses and observer models. This supporting text includes sections on: Translational invariance of subjects' behavior; Success probability; Inverted Gaussian loss function; Model comparison with DIC; Model comparison for different shared parameters between sessions; Nonstationary analysis. (PDF) [file pcbi.1003661.s002.pdf]

On the Origins of Suboptimality in Human Probabilistic Inference

L. Acerbi, S. Vijayakumar and D. M. Wolpert

Supporting Text S1 – Additional analyses and observer models

Contents

1 Translational invariance of subjects' targeting behavior **2**

2 Success probability **3**

3 Inverted Gaussian loss function **4**
3.1 Observer model with variable loss width σ_ℓ 4

4 Model comparison with DIC **5**
4.1 Basic model comparison 5
4.2 Comparison of alternative models of decision making 5
4.3 Comparison of distinct model components 8

5 Model comparison for different shared parameters between sessions **9**

6 Nonstationary analysis **11**
6.1 Iterative non-Bayesian observer model 11

1 Translational invariance of subjects' targeting behavior

In this section we show that subject's behavior depends only on the relative position of the cue with respect to the prior. This result allows us to express all positions in a 'prior-centric' coordinate system ($\mu_{prior} = 0$) without loss of generality.

In the paper we assumed that all variables (e.g. cue position x_{cue}, subjects' response r, target position x) can be expressed relative to the current location of the prior (μ_{prior}); a shift of μ_{prior} simply produces an equal shift in all other position variables. That is, subjects' behavior is independent of screen coordinates (translational invariance). The alternative hypothesis is that subjects' responses instead show some form of bias that is screen-coordinate dependent, for example a central tendency towards the middle of the screen.

In order to test whether subjects' relative responses depend on the absolute location of the prior, for each subject we fit a linear regression line to the relation between the relative response $\tilde{r} = r - \mu_{prior}$ and the prior mean μ_{prior} across all trials. Given the generative model of our task, we expected the average relative response to be zero irrespective of prior mean, $\langle \tilde{r} \rangle = 0$ and therefore tested whether the slope or intercept are different than zero.

For almost all subjects, the slope and intercept were not significantly different than zero ($p > 0.05$). For two subjects we found that slope or intercept may be significantly different from zero ($p = 0.002$ and $p = 0.04$). However, even in these cases a correction for multiple comparisons ($n = 24$) suggests that the these differences are not statistically significant or at most marginally so. This analysis confirms that subjects' responses in general do not show statistically significant departures from the assumption of translational invariance.

2 Success probability

Figure 1 shows the success probability (see Methods in the paper) averaged across subjects, divided by sessions.

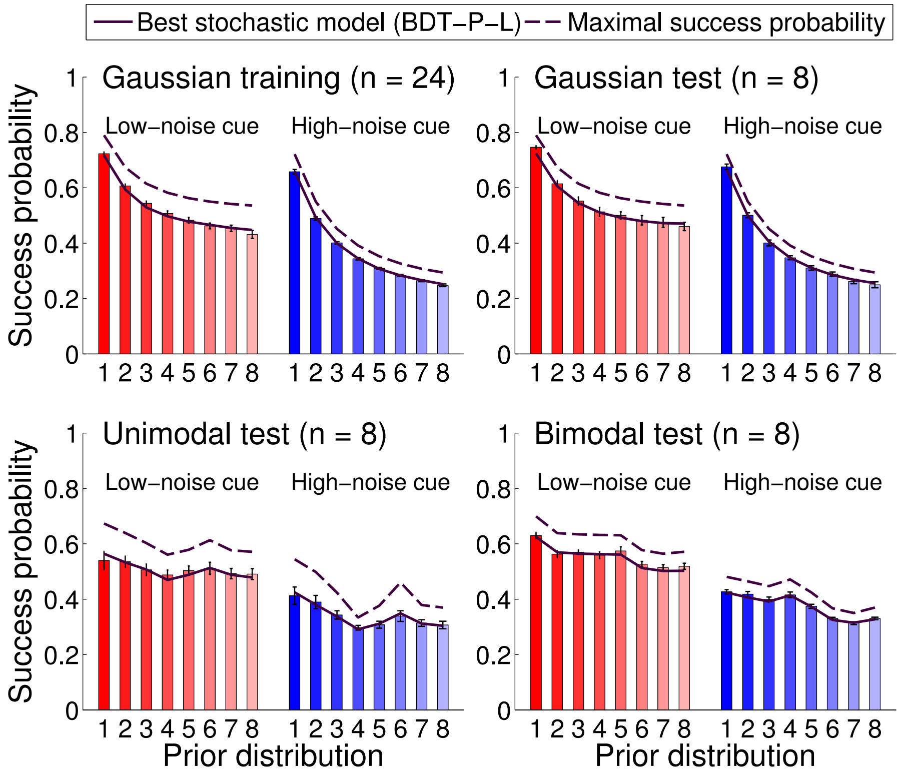

Figure 1. Group mean success probability for all sessions. Each bar represents the group-averaged success probability for a specific session, for each prior (indexed from 1 to 8, see also Figure 2 in the paper) and cue type, low-noise cues (red bars) or high-noise cues (blue bars). Error bars are SE across subjects. Priors are arranged in the order of differential entropy (i.e. increasing variance for Gaussian priors), except for 'unimodal test' priors which are listed in order of increasing width of the main peak in the prior (see main paper). The dashed line represents the maximal success probability for an ideal observer. The continuous line represents the 'postdiction' of the best Bayesian model, BDT-P-L (see 'Analysis of best observer model' in the paper). Also, compare this figure with Figure 5 in the paper, which shows the optimality index.

3 Inverted Gaussian loss function

In this section we show that the inverted Gaussian loss function described by Eq. 16 in the paper is a very good approximation of the true loss model of the task, the square well loss (Eq. 15 in the paper), meaning that in our analysis we can adopt the inverted Gaussian without loss of generality.

In order to compare the Gaussian loss with the square well loss, we first compute the theoretical distribution of observed cues, given each combination of prior and cue (low-noise and high-noise). The distribution of cues is a convolution between the prior and the cue variability, $p(x_{cue}|p_{prior}, d_{cue}) = \int p_{prior}(x)\mathcal{N}\left(x_{cue}|x, \sigma_s^2(d_{cue})\right) dx$. For each combination we calculate the RMSE between the 'optimal target' predicted by the two loss functions for a certain cue position, weighted by cue probability:

$$RMSE(p_{prior}, d_{cue}) = \left\{ \int_{\mathcal{D}} p(x_{cue}|p_{prior}, d_{cue}) \left[x^*_{Gauss}(x_{cue}) - x^*_{well}(x_{cue})\right]^2 dx_{cue} \right\}^{\frac{1}{2}} \tag{S1}$$

where $\mathcal{D}$ is the range of the discrete representation of $p_{prior}(x)$. We exclude from the analysis single-Gaussian priors, as in that case the predicted optimal target is identical for both loss models. We repeat the calculation for a range of values of the scale of the inverted Gaussian, σ_ℓ, while we keep the window size of the square well loss fixed to the 'true' value ($\ell^* = 0.083$ screen units, the cursor diameter).

This procedure allow us to find the value of σ_ℓ for which the inverted Gaussian loss best approximates the true loss function of the task in terms of observable behavior, by minimizing the average RMSE across all our experimental conditions. We find an optimal value of $\sigma_\ell^* \approx 0.027$ screen units, close to the SD of a uniform distribution of range ℓ^*, which is 0.024 screen units (the square well loss can be thought of as an 'inverted uniform distribution'). For σ_ℓ^*, the total RMSE is $1.2 \cdot 10^{-4} \pm 1.5 \cdot 10^{-4}$ screen units (mean $\pm$ SD across different conditions), which is on average less than a tenth of a mm. In terms of performance, the optimality index of an ideal Bayesian observer that uses the inverted Gaussian loss in place of the square-well loss is 0.9999 ± 0.0001 (mean $\pm$ SE across conditions) which is empirically indistinguishable from 1. This analysis shows that the inverted Gaussian loss approximates the behavior of the square well loss far below empirical error for our set of distributions. Hence we can use the inverted Gaussian loss function for our Bayesian observer models without loss of generality.

The inverted Gaussian loss has several advantages over the square well loss. Primarily for us, it allows us to derive an analytic expression of the expected loss that involves only sums of Gaussian distributions (see Eq. 4 in the paper). In general, the inverted Gaussian loss is also a very flexible model, as the scale parameter σ_ℓ allows to interpolate between two very well-known models of loss, a delta function (for $\sigma_\ell \to 0$, which leads to a MAP solution) and a quadratic loss (for $\sigma_\ell \to \infty$, corresponding to the mean of the posterior). In addition to theoretical appeal, experimentally the inverted Gaussian loss has been proven to account very well for people's behavior in a spatial targeting task [1].

3.1 Observer model with variable loss width σ_ℓ

In the paper we either fixed σ_ℓ to the value that best approximates the square well loss or we considered models that explictly or implicitly assume a quadratic loss ($\sigma_\ell \to \infty$). Here we examine the performance of an extended BDT-P-L model (the best model that follows BDT) in which the loss width σ_ℓ is allowed to vary freely. Since the parameter σ_ℓ is irrelevant for Gaussian posteriors, we perform this analysis only for non-Gaussian posteriors (see Methods in the paper). Given the typical scale of the posteriors in the task, a value of $\sigma_\ell \gtrsim 0.2$ screen units should be considered near-quadratic for all practical purposes.

We find that subjects fall in two classes with respect to the posterior distribution of parameter σ_ℓ. For the majority of subjects (10 out of 16), mostly in the bimodal session, the posterior is peaked around $\sigma_\ell = 0.11 \pm 0.02$ screen units (mean $\pm$ SE across subjects), which is significantly higher than the 'true' value ($\sigma_\ell^* = 0.027$ screen units; signed rank test, $p < 0.01$) but still qualitatively different from a near-quadratic loss. For the other six subjects the posterior is much broader and flat in the range of σ_ℓ

from 0.2 to 1 screen units, compatibly with a near-quadratic loss. In fact, according to the comparison between alternative models of decision making, these subjects show some preference for a quadratic loss or, similarly, a low-order approximation of the posterior (see Figure 9a in the paper and Figure 3a here, subjects 10-14 and 18). However, note that most of these subjects belong to the unimodal group, where posteriors are still very close to Gaussians and therefore the exact value of the loss width may not be necessarily meaningful. The reason why we find a relatively large loss width in the case of a BDT observer is that it needs to account for large, posterior-dependent targeting errors that are explained instead by stochasticity in decision making by the SPK observer (in neither case posterior-dependent errors can be adequately explained by constant motor noise σ_{motor}).

Performance of model BDT-P-L with variable loss is better than its corresponding version with fixed σ_ℓ (ΔDIC $= -11.5 \pm 4.0$, $p < 0.05$), but still slightly worse than a model with variability in decision making with the same number of parameters, SPK-L (ΔDIC $= 22.5 \pm 8.9$, $p < 0.05$). In conclusion, allowing a degree of freedom to the loss function at most slightly improves model performance for BDT but does not seem to provide a better explanation for the data than models with variability in decision making.

4 Model comparison with DIC

We report in this section the DIC scores of invidual models for all subjects, and results of the group DIC (GDIC) model comparison. DIC scores are used in the paper to approximate the marginal likelihood of each dataset and model within a hierarchical Bayesian model selection (BMS) framework [2]. Here we also use DIC scores to compute the average impact of each model factor.

4.1 Basic model comparison

Figure 2a shows the model evidence for each individual model and subject. We calculated model evidence as the difference in DIC between a given model and the subject's best model (lower values are better). A difference of more than 10 in this scale should be considered strong evidence for the model with lower DIC. Individual results show that model SPK-P-L performed better than other models for almost all datasets, with the exception of a minority that favored model SPK-P instead. Unlike our BMS analysis, here we see a considerable similarity of performance between model SPK-P-L and SPK-S-P-L, although the latter performs slightly worse than the former in almost all cases. Figure 2b shows the group average DIC (GDIC), relative to the model with lowest average DIC (lower scores are better). SPK-P-L is confirmed as the best model. Model SPK-S-K-L comes second in terms of average score, but note that the difference with SPK-P-L is very significant (pairwise signed-rank test with Bonferroni correction for multiple comparisons, $p < 0.001$). This suggests that the extra model factor S is not improving model performance, and therefore that SPK-S-P-L is not a 'good' model, in agreement with the small support it obtained in the BMS analysis (see main paper).

4.2 Comparison of alternative models of decision making

We consider first the model evidence for each individual model and subject (Figure 3a). Results differ depending on the session (unimodal or bimodal). In both sessions model SPK-L performs consistently well, closely followed by model SPK. However, in the unimodal session there are quite a few subjects whose behavior is well described by several other models. These results are summarized in Figure 3b, which shows the group DIC relative to the model with lowest average DIC (lower scores are better). Due to the difference between sessions we separately computed the group averages for the unimodal and bimodal group. GDIC analysis in the unimodal session alone fails to find significant differences between

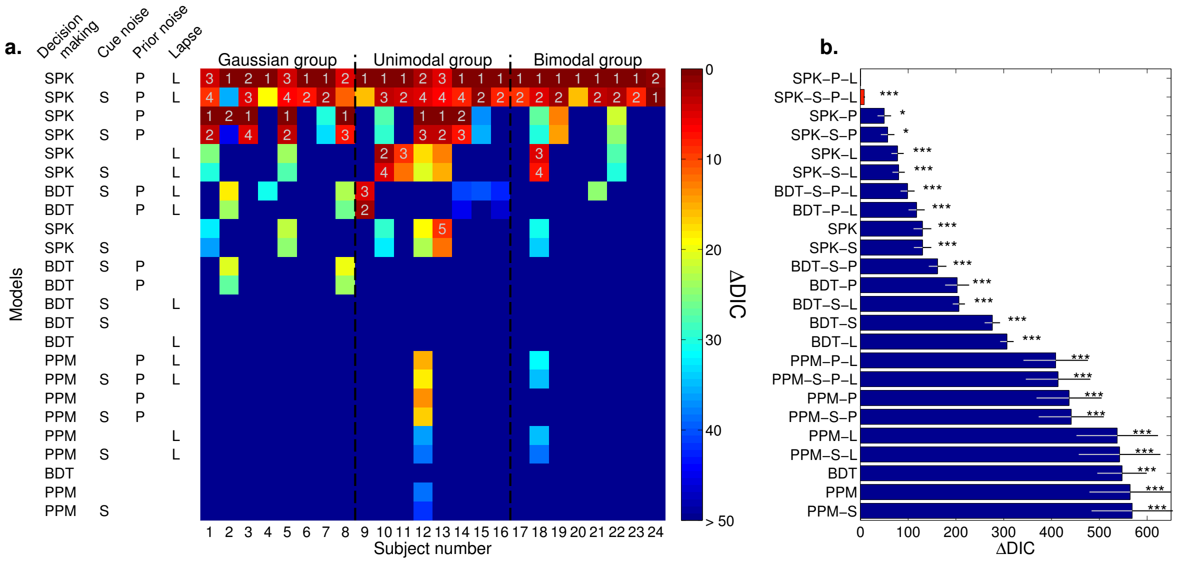

Figure 2. Model comparison between individual models (DIC scores). a: Each column represents a subject, divided by test group (all datasets include a Gaussian training session), each row an observer model identified by a model string (see Table 2 in the paper). Cell color indicates model's evidence, here displayed as the DIC difference (ΔDIC) with the best model for that subject (a higher value means a worse performance of a model with respect to the best model). Models are sorted by their group average DIC score (see panel b). Numbers above cells specify ranking for most supported models with comparable evidence (ΔDIC less than 10). **b:** Group average ΔDIC score, relative to the best model (mean ± SE). Higher scores indicate worse performance. Asterisks denote significant difference in DIC between a given model and the best model, after correction for multiple comparisons: (*) $p < 0.05$, (***) $p < 0.001$.

SPK-L and several other observer models. Conversely, GDIC shows significant results in the bimodal session, finding that all models but SPK perform worse than SPK-L.

These results agree with the BMS analysis in the paper in indicating SPK-L as the best model, but otherwise present quite a different pattern. Discrepancies between the two model comparison methods emerge for the following reasons. Firstly, as mentioned in the paper, BMS is not affected by outliers and by construction takes into account group heterogeneity, contrarily to DIC. Secondly, posteriors in the unimodal session may still be very close to Gaussian and therefore distinct models share very similar predictions, which DIC scores alone cannot disambiguate. The hierarchical probabilistic structure of BMS, instead, allows information to flow between global model evidence and individual model evidence for each subject (respectively α and u_{nk} in [2]), at each iteration of the model comparison algorithm. This propagation of belief led BMS to discard less likely models in the paper.

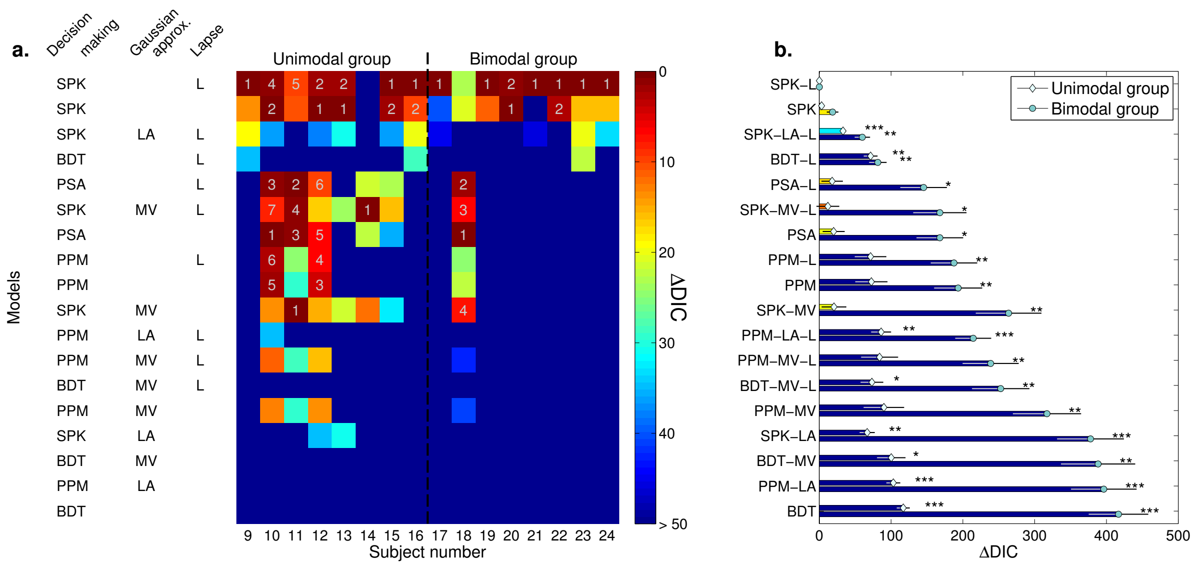

Figure 3. Model comparison between alternative models of decision making (DIC scores). We tested a class of alternative models of decision making which differ with respect to predictions for non-Gaussian trials only. **a:** Each column represents a subject, divided by group (either unimodal or bimodal test session), each row an observer model identified by a model string (see Table 2 in the paper). Cell color indicates model's evidence, here displayed as the DIC difference (ΔDIC) with the best model for that subject (a higher value means a worse performance of a model with respect to the best model). Models are sorted by their group average DIC score across both sessions (see panel b). Numbers above cells specify ranking for most supported models with comparable evidence (ΔDIC less than 10). **b:** Group average ΔDIC, divided by test group (unimodal or bimodal session), relative to the best model (mean $\pm$ SE). Higher scores indicate worse performance. Asterisks denote significant difference in DIC between a given model and the best model, after correction for multiple comparisons: (*) $p < 0.05$, (**) $p < 0.01$, (***) $p < 0.001$.

4.3 Comparison of distinct model components

We assess the relevance of each model level within a factor by measuring the average contribution to DIC of each level across all tested observer models, relative to the best level (Figure 4). This is the GDIC counterpart of the BMS computation of the posterior likelihood of each model component (Figures 8c and 9c in the paper). Results of the GDIC analysis are qualitatively similar to BMS for all factors, with the sole exception of factor S (sensory noise in estimation of the cue position). BMS rejects factor S, whereas from GDIC we can see that, on average, it seems that *not* having factor S decreases model performance (ΔDIC: 33.0 ± 5.6, mean $\pm$ SE across subjects). This is not a contradiction: for many simple observer models the addition of any reasonable form of noise, including cue-estimation noise, will improve model performance. However, model factor S becomes redundant when other more fitting forms of noise are present. Since GDIC weights equally all model contributions, model S appears to have a useful influence on model performance due to the average contribution of 'simpler' models. On the contrary, BMS weights evidence differentially and component S appears to be irrelevant for the most likely models (see paper).

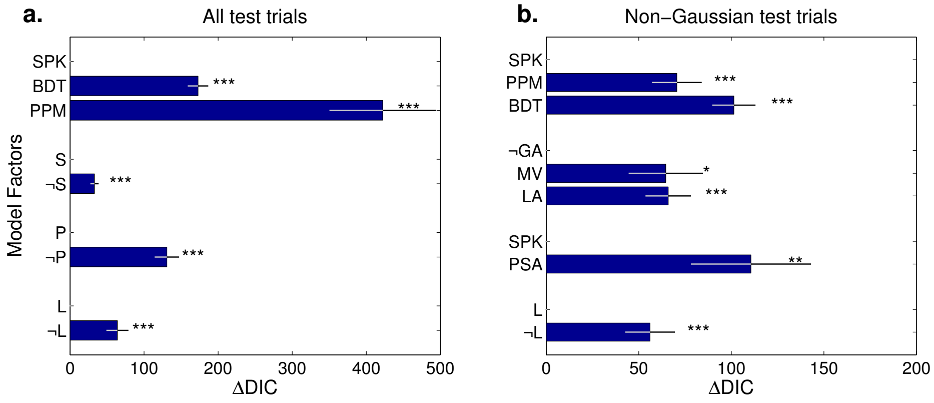

Figure 4. Influence of different model factors on DIC. Difference in DIC between different levels within factors, relative to the best level (lowest DIC); highest scores denote worse performance. Each group of bars represent a factor, each bar a level within the factor, identified by a model label (see Table 2 in the paper). Error bars are SE across subjects. Asterisks denote significant difference in DIC between a given level and the best level, after correction for multiple comparisons. **a:** Factors in the basic model comparison. **b:** Factors in the comparison of alternative models of decision making. Label '¬GA' stands for no Gaussian approximation (full posterior).

5 Model comparison for different shared parameters between sessions

In the paper we assumed that each subject shared two parameters between the training session and the test session (the motor noise σ_{motor} and the ratio between the cue noise, $\tilde{\sigma}_{high}/\tilde{\sigma}_{low}$), whereas all the other parameters were specified separately for the two sessions (see 'Model comparison' section in the paper). Here we motivate our modelling choice by showing that it is optimal, at least on a subset of observer models. By 'optimal' we mean that models that share more parameters between sessions perform substantially worse, whereas models that share less parameters (and therefore have more free parameters to specify) do not provide a significant advantage.

For the current analysis we consider a set of variants of observer model SPK (stochastic posterior). We focus on this model since it is the simplest model with the 'best' decision-making component, as found in the paper. These variants differ from the standard SPK model only with respect to the number of parameters shared between training and test sessions. For a single session, model SPK can be characterized by four parameters ($\sigma_{motor}, \tilde{\sigma}_{low}, \tilde{\sigma}_{high}, \kappa$; see 'Suboptimal Bayesian observer models' section in the paper). Table 1 lists the considered variants, labelled by number of parameters shared across sessions (model SPK#2 corresponds to the variant adopted in the paper).[1]

Model	Total number of parameters	Free parameters ($\boldsymbol{\theta}_M$)
SPK#4	4	$\sigma_{motor}, \tilde{\sigma}_{low}, \tilde{\sigma}_{high}, \kappa$
SPK#3	5	$\sigma_{motor}, \tilde{\sigma}_{low}, \tilde{\sigma}_{high}, \kappa \times 2$
SPK#2	6	$\sigma_{motor}, \tilde{\sigma}_{low}, (\tilde{\sigma}_{high}, \kappa) \times 2$
SPK#1	7	$\sigma_{motor}, (\tilde{\sigma}_{low}, \tilde{\sigma}_{high}, \kappa) \times 2$
SPK#0	8	$(\sigma_{motor}, \tilde{\sigma}_{low}, \tilde{\sigma}_{high}, \kappa) \times 2$

Table 1. Observer model SPK with different shared parameters. Table of observer models based on SPK (stochastic posterior) but with different number of shared parameters (model SPK#2 corresponds to the version in the paper). The number after the '#' symbol represents the number of parameters the model shares between training and test session. For each model it is also specified the total number of free parameters used to characterize both sessions. A '×2' means that a parameter is specified independently for training and test sessions; otherwise parameters are shared across sessions. See main text and Methods in the paper for the meaning of the various parameters.

Here we use GDIC instead of BMS since we want to find the modelling choice that works best on average for all subjects. Figure 5 shows the relative DIC scores of the model for different number of shared parameters. Unsurprisingly, the model with lowest group DIC is the model with the highest number of parameters (SPK#0). However, models SPK#1 and SPK#2 closely match the performance of model SPK#0. In particular, the difference between SPK#2 and SPK#0 is nonsignificant (ΔDIC = 3.5 ± 2.1; $p = 0.55$). Conversely, observer models with 3 or more shared parameters perform significantly worse (e.g., for SPK#3: ΔDIC = 32.4 ± 7.3; $p < 0.001$).

These results show that a model that shares the motor noise parameter and the ratio between the estimated cues' SDs between sessions achieves the optimal balance between model fit and simplicity, supporting our choice in the paper.

[1] Although there are in total 2^4 variants of model SPK that share different combinations of parameters between sessions, the five models in Table 1 represent the most natural combinations, in order of increasing model complexity.

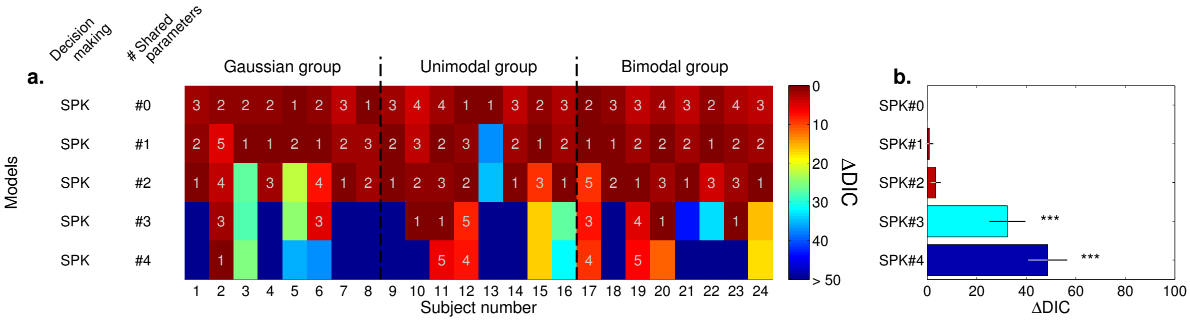

Figure 5. Comparison of models with different number of shared parameters. Model comparison between observer models based on model SPK but with different number of shared parameters between sessions. **a:** Each column represents a subject, divided by test group (all datasets include a Gaussian training session), each row an observer model identified by a model string (see Table 1). Cell color indicates model's evidence, here displayed as the DIC difference (ΔDIC) with the best model for that subject (a higher value means a worse performance of a model with respect to the best model). Models are sorted by their group average DIC score (see panel b). Numbers above cells specify ranking for most supported models with comparable evidence (ΔDIC less than 10). **b:** Group average ΔDIC score, relative to the best model (mean ± SE). Higher scores indicate worse performance. Asterisks denote significant difference in DIC between a given model and the best model, after correction for multiple comparisons: (***) $p < 0.001$.

6 Nonstationary analysis

In our analysis of the data in the paper we have assumed stationarity of participants' behavior: in first approximation, trials are statistically independent and observers' parameters do not change during the course of a session. Stationarity is a common simplifying assumption in the analysis of psychophysical data, although deviations from stationarity can lead to misestimation of the participants' parameters [3]. A typical source of nonstationarity is 'memory', the influence of recent trials on the current response [4]. This is of prime interest to our study, as it could be the case that the variability that we observe in decision making is not random but due to recency effects.

As a simple, model-free test for recency effects, we look at correlations between trial variables at trial i and trial $i+1$. In particular, we define the *error* at trial i, $\text{Error}(i)$, as the difference between the subjects's response r and the true target position x. The *shift* at trial i, $\text{Shift}(i)$, is the difference between subject's response r and the cue position x_{cue}. In formulas:

$$\text{Error}(i) = r^{(i)} - x^{(i)}, \qquad \text{Shift}(i) = r^{(i)} - x^{(i)}_{cue}. \tag{S2}$$

Note that subjects explicitly knew the error only during the training session, in which they received full performance feedback. During the test trials they only received a qualitative feedback on whether they succeded or missed in the trial.

For each subject we analyze separately training and test sessions, computing the correlations between Error and/or Shift between trial i and trial $i+1$ for each dataset ($n = 24$ training sessions and $n = 24$ test sessions, for four possible combinations of variable interaction). Figure 6 shows the trial to trial correlations for individual subjects and their mean. In all cases we find a small but statistically significant anticorrelation between trial variables in the training sessions (t-test, $p < 0.05$) and no significant correlation in the test sessions. The anticorrelation in the training sessions could easily emerge from a strategy that produces small adjustments in the opposite direction of the experienced error vector. Since the test sessions did not provide full feedback, we do not see any significant effect. These small and null effects suggest that the major variability in the subjects' responses, observed in both training and test sessions, was not due to some trivial trial-to-trial correlation.

6.1 Iterative non-Bayesian observer model

Although the correlations seen in Figure 6 are modest, it may be that an iterative (trial-to-trial) model that captures longer-term correlations may fare better at explaining the data. Iterative Bayesian models have been successful at explaining subjects' perfomance in different domains, such as target estimation [5], distance perception [6] and motor adaptation [7]. Simple heuristics may reproduce a behavior that is very close to the Bayesian prediction [4, 8]. We consider here an iterative, non-Bayesian linear observer model with lapse (IT-L) that implements a simple trial-to-trial heuristic.

In a trial without lapse, the non-Bayesian iterative observer chooses the target x according to a linear mapping f of the current cue position x_{cue} (in prior-centric coordinates), depending on the current cue type d_{cue} and prior p_{prior}:

$$x = f\left(x_{cue}; d_{cue}, p_{prior}\right) = W(d_{cue}, p_{prior}) \cdot x_{cue}, \tag{S3}$$

where W is a table of linear weights with one entry for each combination of prior and cue type. The table W is updated on a trial by trial basis according to the feedback received each trial (see below for implementation details). To account for mistakes and other sources of variability, we include a probability of lapse, according to model factor L (see paper) . The final response is as usual obtained by adding motor noise with SD σ_{motor}. Although conceptually simple, the model has a total of eight free parameters, most of which are involved in the update rule in order to allow for maximum flexibility (see below).

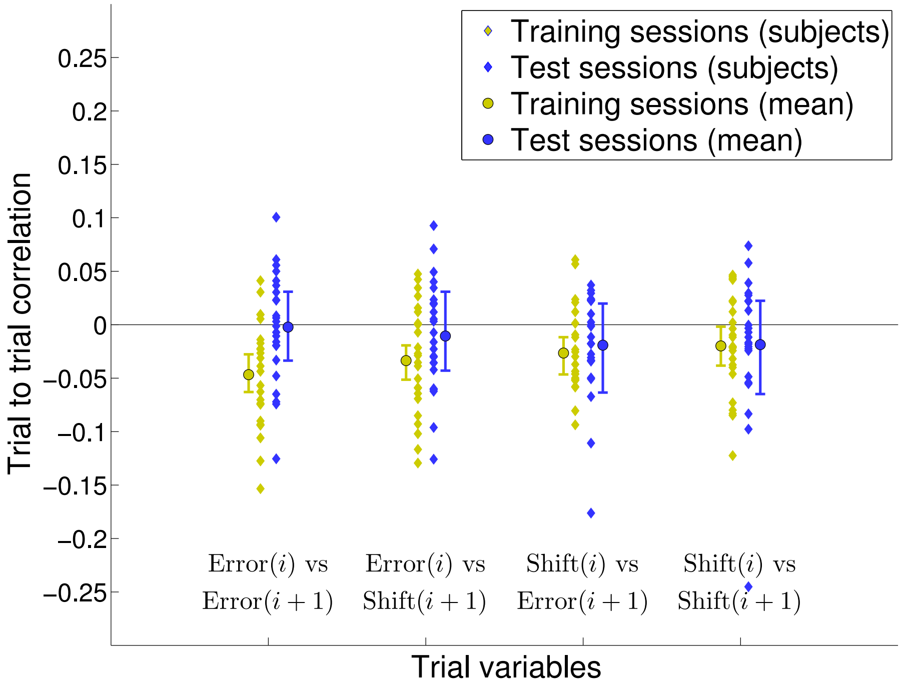

Figure 6. Trial to trial correlations between Error and Shift. Correlations between trial variables at trial i and trial $i+1$ for four possible combinations of relevant variables Error (difference between response and target position) and Shift (difference between response and cue position). Each data point is an individual session (training sessions in green, test sessions in blue). Mean correlations, averaged across subjects, are plotted as circles. Error bars are 95% confidence intervals, computed via bootstrap.

Since in our previous analysis only training sessions showed significant trial-to-trial correlations and, moreover, our update rule assumes that full feedback is available to the subjects, we test the model on the training sessions only. We compare the non-Bayesian iterative model against model SPK (stochastic posterior), the simplest Bayesian observer that includes variability in decision making. GDIC analysis shows that model SPK significantly outperforms model IT-L (20 subjects out of 24; paired signed-rank test $p < 0.01$). We, therefore, reject the hypothesis that our data can be explained by this simple iterative non-Bayesian model (see Discussion in the paper).

Implementation of the model

For model IT-L, we substitute Eq. 11 of the paper with a trial-dependent equation,

$$p_{target}^{(lapse)}\left(x^{(i)}|x_{cue}^{(i)}, d_{cue}^{(i)}, p_{prior}^{(i)}\right) = (1-\lambda)\cdot\delta\left[x^{(i)} - f\left(x_{cue}^{(i)}, d_{cue}^{(i)}, p_{prior}^{(i)}\right)\right] + \lambda\cdot p_{prior}^{(i)}\left(x^{(i)}\right) \qquad \text{(S4)}$$

where all variables now show a dependence on the trial number i, but otherwise all symbols have a comparable role as in Eq. 11 in the paper. Here, $f\left(x_{cue}, d_{cue}, p_{prior}\right)$ is assumed to be a linear mapping from the position of the cue to the chosen target (see Eq. S3), whose linear weights are stored in table $W^{(i)}$, which is updated each trial. The table contains a separate entry for each combination of prior type (p_{prior}, or equivalently σ_{prior} for Gaussian priors) and cue type (d_{cue}, either 'short' or 'long'). We assume for simplicity that the table is initialized with two weight values, respectively one for all short, low-noise

cues, $w^{(0)}_{short}$, and another one for long, high-noise cues, $w^{(0)}_{long}$, irrespective of prior type.

In a noise-free scenario, in each trial the error term between current weight and 'correct' weight (according to feedback) can be computed as:

$$\delta^{(i)} = \frac{r^{(i)}}{x^{(i)}_{cue}} - \frac{x^{(i)}}{x^{(i)}_{cue}} = \left(r^{(i)} - x^{(i)}\right) \frac{1}{x^{(i)}_{cue}} \tag{S5}$$

where $x^{(i)}$ is the actual target position (all positions are measured in coordinates relative to the mean of the prior). However, due to noise, Eq. S5 can take arbitrarily large values because of $x^{(i)}_{cue}$ at the denominator. We therefore apply a regularization factor to the error, so that

$$\delta^{(i)} = \left(r^{(i)} - x^{(i)}\right) \cdot \frac{\operatorname{sgn}\left(x^{(i)}_{cue}\right)}{\left|x^{(i)}_{cue}\right| + \omega}; \tag{S6}$$

with $\omega > 0$. For the update rule we take a delta-rule [8]:

$$W^{(i+1)}\left(\sigma_{prior}, d_{cue}\right) = W^{(i)}\left(\sigma_{prior}, d_{cue}\right) - \eta \cdot \delta^{(i)} \cdot g\left(\sigma_{prior}, d_{cue}, \sigma^{(i)}_{prior}, d^{(i)}_{cue}\right) \tag{S7}$$

where $\eta > 0$ is a learning factor and $g\left(\sigma_{prior}, d_{cue}, \sigma'_{prior}, d'_{cue}\right)$ a transfer function assessing how the learning about a specific combination of prior and cue generalizes to another combination. We assume a simple local learning of the form

$$g\left(\sigma_{prior}, d_{cue}, \sigma'_{prior}, d'_{cue}\right) = e^{-\frac{\left(\sigma_{prior} - \sigma'_{prior}\right)^2}{2\Delta^2_{\sigma}}} \cdot e^{-\frac{\left(d_{cue} - d'_{cue}\right)^2}{2\Delta^2_{cue}}} \tag{S8}$$

where Δ_{σ} and Δ_{cue} are two parameters measuring the generalization length respectively in prior and cue space. Overall, the model has eight parameters: the motor variability σ_{motor} and the lapse rate λ, the initial weights $w^{(0)}_{short}$ and $w^{(0)}_{long}$, the learning factor η, the regularization parameter ω and the generalization lengths Δ_{σ} and Δ_{cue}.

This wide array of parameters allows the model to capture different possible classes of non-Bayesian strategies and update rules. Given the number of parameters and complexity of the log likelihood space of the model, when computing the posterior distribution of the parameters we ran much longer chains in order to improve convergence of the sampling algorithm ($3 \cdot 10^5$ burn-in samples, $3 \cdot 10^5$ saved samples per chain).

References

1. Körding KP, Wolpert DM (2004) The loss function of sensorimotor learning. Proc Natl Acad Sci U S A 101: 9839–9842.

2. Stephan KE, Penny WD, Daunizeau J, Moran RJ, Friston KJ (2009) Bayesian model selection for group studies. Neuroimage 46: 1004–1017.

3. Fründ I, Haenel NV, Wichmann FA (2011) Inference for psychometric functions in the presence of nonstationary behavior. J Vis 11: 1–19.

4. Raviv O, Ahissar M, Loewenstein Y (2012) How recent history affects perception: the normative approach and its heuristic approximation. PLoS Comput Biol 8: e1002731.

5. Berniker M, Voss M, Kording K (2010) Learning priors for bayesian computations in the nervous system. PLoS One 5: e12686.

6. Petzschner F, Glasauer S (2011) Iterative bayesian estimation as an explanation for range and regression effects: a study on human path integration. J Neurosci 31: 17220–17229.

7. Verstynen T, Sabes PN (2011) How each movement changes the next: an experimental and theoretical study of fast adaptive priors in reaching. J Neurosci 31: 10050–10059.

8. Nassar MR, Wilson RC, Heasly B, Gold JI (2010) An approximately bayesian delta-rule model explains the dynamics of belief updating in a changing environment. J Neurosci 30: 12366–12378.
