# Supplementary material for: On the Origins of Suboptimality in Human Probabilistic Inference
Source: PLoS Comput Biol. 2014 Jun 19;10(6):e1003661. doi: 10.1371/journal.pcbi.1003661 (PMC4063671; doi:10.1371/journal.pcbi.1003661)
Supplement: Text S2 — Noisy probabilistic inference. Description of the models of stochastic probabilistic inference (‘noisy posterior’ and ‘sample-based posterior’) and discussion about unstructured noise in the prior. (PDF) [file pcbi.1003661.s003.pdf]

On the Origins of Suboptimality in Human Probabilistic Inference

L. Acerbi, S. Vijayakumar and D. M. Wolpert

Supporting Text S2 – Noisy probabilistic inference

We introduce two alternative models of stochastic computations in Bayesian inference ('Stochastic posterior models'). The first one (noisy posterior) comprises a representation of the posterior corrupted by noise; in the second one (sample-based posterior), a discrete, approximate representation of the posterior distribution is built out of a number of samples drawn from the posterior.

We show that, for the loss function of our task, for both models the predicted distribution of chosen targets is quantitatively very close to a power function of the posterior distribution in the trial ('Results'). The generality of this result motivates the power function approximation used for decision-making model level SPK (stochastic posterior), Eq. 7 in the paper.

Lastly, we show that, under specific assumptions, the stochasticity in the posterior can also represent a certain type of noise in the prior ('Stochastic posterior from unstructured noise in the prior').

Stochastic posterior models

According to Bayesian Decision Theory (BDT), the computation of the optimal target x^* for a given loss function $\mathcal{L}$ requires three steps:

1. Computation of the posterior probability $p_{post}(x)$.
2. Computation of the expected loss, $\mathcal{E}(x) = \int p_{post}(x)\mathcal{L}(x, x')dx'$.
3. Computation of the target x^* that minimizes the expected loss, $x^* = \arg\min_x \mathcal{E}(x)$.

Step 1 corresponds to the *inference* step and is described by Eq. 2 in the paper. Steps 2 and 3 correspond to *action selection* (Eq. 4 in the paper).

In principle, noise in decision making could be added to any of the above steps. For parsimony, here we consider models that adds stochasticity to the computation (or representation) of the posterior distribution (step 1), and we analyze how this noise propagates to the inferred optimal target x^*. However, our results are compatible also with noise injected at later stages (e.g. in action selection).

Noisy posterior

For ease of calculation, we convert the continuous posterior distribution $p_{post}(x)$ to a discrete probability distribution $p_i = p_{post}(x_i)$ for a discrete set of target values $\{x_i\}_{1 \le i \le N}$ where we assume that the x_i cover uniformly the target space with dense spacing Δx.[1]

[1] The discretization step could be skipped by modelling continuous noise with a Gaussian process [1]. However, the discrete representation makes the model simpler and easier to interpret. The lattice spacing Δx is related to the correlation length of a Gaussian process and affects the amount of noise and discretization error.

We model the computation of a 'noisy posterior' (step 1) by adding normally distributed noise to the posterior (see Figure 7b in the paper):

$$\widetilde{p}_{post}(x) = \sum_{i=1}^{N} y_i \delta\left(x - x_i\right) \qquad \text{with} \quad y_i = p_i + \sigma(p_i)\eta_i \tag{S1}$$

where the η_i are i.i.d. normal random variables and $\sigma(p_i)$ is the SD of the 'decision noise', that in general depends on the value p_i.[2] For simplicity, the η_i are assumed to be statistically independent but it is easy to extend the model to take into account correlations in the noise.

For the form of $\sigma(p)$ we consider two common alternative rules:

- A Poisson-like law: $\sigma_{Poisson}(p) = \sqrt{p/g}$, where we have defined $g > 0$ as a 'neuronal gain' parameter; higher gain corresponds to less noise. The rationale for this rule is that the y_i can be thought of as a population of N independent units or channels ('neurons'), each one noisily encoding the posterior probability at a given target value x_i (see Figure 7b in the paper). The activation of each unit ('firing rate'), with a global rescaling factor g, takes the form $y_i = gp_i + \sqrt{gp_i}\eta_i$ which approximates the response of a Poisson neuron with mean activation gp_i.
- Weber's law (multiplicative noise), in which the noise is proportional to the probability itself, a form of variability which is typical to many sensory magnitudes: $\sigma_{Weber}(p) = w \cdot p$, with $w > 0$ the *Weber's fraction.*

For a fixed lattice spacing Δx, this model of noise in decision making has only one free parameter, g (or w), that sets the amount of variability in the inference. Note that the 'neural population' description allows for an intuitive understanding of Eq. S1, but the noisy posterior model does not require to commit to this intepretation.

Sample-based posterior

This model assumes that a discrete, approximate representation of the posterior is constructed by drawing K samples from the posterior [2–4] (see Figure 7c in the paper):

$$\widetilde{p}_{post}(x) = \frac{1}{K} \sum_{i=1}^{K} \delta\left(x - x^{(i)}\right) \qquad \text{with} \quad x^{(i)} \sim p_{post} \tag{S2}$$

where the $x^{(i)}$ are i.i.d. samples from the posterior. The parameter K is inversely proportional to the noise in the representation.

Target choice distribution

For a given posterior distribution $p_{post}(x)$, Eqs. S1 and S2 allow us to compute several instances of a stochastic posterior $\widetilde{p}_{post}(x)$ which, after minimization of the expected loss, entail different chosen targets x^*. By repeating this procedure and binning the results, we can obtain the shape of the distribution of target choices $p_{target}(x)$ for a given model of stochasticity (see Figure 7e & 7f in the paper). However, this method is computationally very expensive.

A simple expression for $p_{target}(x)$ is needed in order to make efficient use of a stochastic posterior model in data analysis, e.g. to compute the marginal likelihood of a dataset. Our goal is to show that the

[2] Formally, $\widetilde{p}_{post}(x)$ as defined in Eq. S1 is not a probability distribution since, aside of normalization, it is not always non-negative (the p_i's may take negative values for large amounts of noise in the inference). In this case the 'noisy posterior' should be more correctly interpreted simply as an intermediate step in a noisy computation of the expected loss.

target choice probability of these noisy decision-making models is well approximated by a power function of the posterior distribution:

$$p_{target}(x) \sim [p_{post}(x)]^{\kappa} \tag{S3}$$

where $\kappa \geq 0$ is an appropriate exponent that is the direct equivalent of the noise parameter g, w or K; higher values of κ correspond to less decision noise. In general, we would like the exponent in Eq. S3 to be a function of the noise parameter, that is for example $\kappa = \kappa(g)$, where the mapping does not depend on the posterior distribution itself but only on the decision noise level (note that the mapping will depend on other fixed details of the model such as the loss function, and the chosen discretization spacing Δx for the 'noisy posterior' model).

Results

We computed the target choice probability predicted by the stochastic posterior models in our task (noisy posterior with either Poisson-like or Weber's law noise, and sample-based posterior). We chose as loss function the inverted Gaussian approximation used by the observer models in the paper (see Methods in the paper; results did not qualitatively change with the square well loss). We took as posterior distributions a representative set of all posterior distributions of the task, built out of several combinations of prior, cue position and cue type (low-noise and high-noise cues), for a total of about 1000 posterior distributions. We took several levels of decision noise (values of g, w or K, depending on the model), ranging from an approximately correct inference to an extremely noisy inference. For each posterior distribution and decision noise level we calculated the shape of the target choice distribution via Monte Carlo sampling (10^5 samples per distribution).

Figure 1 shows the target choice distributions and related posterior-power fit distributions (Eq. S3) for three illustrative posteriors and five levels of decision noise for the noisy posterior model with Poisson-like noise. For high levels of decision noise, the target choice distribution resembles the posterior distribution (i.e. a posterior-matching strategy), whereas for low levels of decision noise it becomes a narrow distribution peaked on the mode of the posterior (the model tends to a MAP strategy for $g \to \infty$). This may intuitively explain why a power function of the posterior would be a good approximation of the target choice distribution.

We quantified how well a power function of the posterior can approximate the target choice distributions in terms of Kullback-Leibler (KL) divergence. For each noise level, we computed the exponent κ that minimizes the KL divergence between posterior-power distributions and target choice distributions in the set (crucially, the same exponent κ fit simultaneously all $\sim$ 1000 distributions). To assess the goodness of fit in our experiment, we computed mean and SD of the KL divergence according to a log-normal approximation of the posterior distribution of values of κ found in the test sessions for our subjects (see paper, section 'Analysis of best observer model').

In general, we found that the posterior-power fit approximates quite well the target choice distribution of all stochastic posterior models. The KL divergence between true distribution and its approximation was $\sim 0.02 \pm 0.02$ nats (mean $\pm$ s.d. across the distribution of values of κ) for all distinct models of noisy inference. These values are equivalent to the KL divergence between two Gaussian distributions with same SD and whose means differ by about one-fourth of their SD.

This analysis shows that a power function of the posterior represents a good approximation of the distribution of target choices of a Bayesian oberver that takes action according to a noisy or sample-based representation of the posterior. This result provides a sound basis for the analytical form chosen for model level SPK (stochastic posterior), Eq. 7 in the paper.

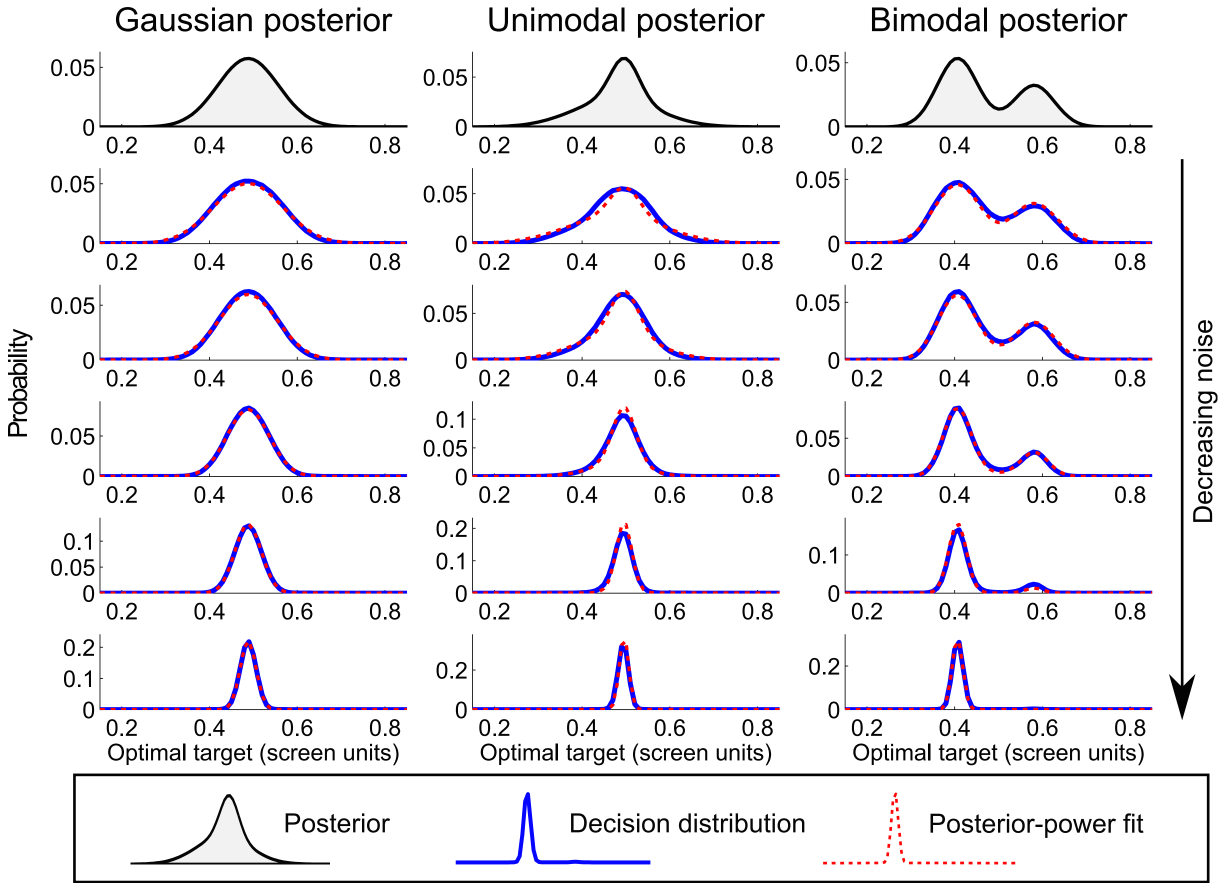

Figure 1. Posterior-power approximation of the noisy posterior model. Comparison between the target choice distributions computed according to the true noisy posterior model (with Poisson-like noise) and their posterior-power approximations. The various panels show the target choice distributions $p_{target}(x)$ (blue lines) and the associated posterior-power fits (red dashed lines) for different posterior distribution and noise level g in the computation. Each column corresponds to a different illustrative posterior distribution, shown on top, divided by class (Gaussian, unimodal and bimodal). Each row, excluding the first, corresponds to a different level of decision noise, with noise decreasing from top to bottom. Analogous fits were found for the sample-based approximation of the posterior.

Stochastic posterior from unstructured noise in the prior

We show here that the posterior noise model SPK may also subsume the unstructured components of noise in the prior.

If we assume that the internal measurement of the prior is corrupted by multiplicative sensory noise (according to the approximate Weber's law for density or numerosity esimation [5]) and that it changes smoothly in the target position, the estimated prior can be written as:

$$\tilde{p}_{prior}(x) = p_{prior}(x) \cdot (1 + \epsilon(x)) \tag{S4}$$

where $\epsilon(x)$ is a Gaussian process with zero mean and some appropriately chosen SD and covariance function (see [1]). Crucially, if the observer uses Eq. S4 to build a posterior distribution, we obtain:

$$\tilde{p}_{post}(x) = p_{post}(x) \, (1 + \epsilon(x)) \tag{S5}$$

where $p_{post}(x)$ is the usual, non-noisy posterior (Eq. 2 in the paper). Eq. S5, once appropriately discretized, is formally equivalent to the equation we used to describe a noisy posterior with multiplicative noise (Eq. S1; see also Figure 7b in the paper). Therefore, under these assumptions, the random, unstructured components of noise in the prior can be absorbed within the noisy posterior model.

Note that the estimation noise on the prior that we considered in the paper, model factor P, is a structured form of noise that varies along task-relevant dimensions (such as the width of the prior or the relative weights of bimodal priors). Whereas structured noise can be identified at least in principle, teasing out which stage or component unstructured noise belongs to represents a greater challenge. For example, an experiment that involves a variable number of inference step may be able to distinguish whether noise stems from the computation of the posterior, which is repeated at every step, or from noise in the encoding of the original prior, which happens only once. A paradigm of this kind has been recently used to explore similar issues in a perceptual categorization task [6]. However, this method is still unable to distinguish whether noise appears in the first step (in the encoding or recall of the prior) or at the very last stage, during action selection. Another way to identify noise in the prior could consist in imposing a strong hyperprior on the subjects via extensive training. The level of attraction to such hyperpriors, once learned, may be indicative of the amount of uncertainty in the subjects' measurement of the prior.

References

1. Rasmussen C, Williams CKI (2006) Gaussian Processes for Machine Learning. The MIT Press.

2. Sundareswara R, Schrater PR (2008) Perceptual multistability predicted by search model for bayesian decisions. J Vis 8: 1–19.

3. Vul E, Goodman ND, Griffiths TL, Tenenbaum JB (2009) One and done? optimal decisions from very few samples. In: Proceedings of the 31st annual conference of the cognitive science society. volume 1, pp. 66–72.

4. Fiser J, Berkes P, Orbán G, Lengyel M (2010) Statistically optimal perception and learning: from behavior to neural representations. Trends Cogn Sci 14: 119–130.

5. Ross J (2003) Visual discrimination of number without counting. Perception 32: 867–870.

6. Drugowitsch J, Wyarta V, Koechlin E (2014). The origin and structure of behavioral variability in perceptual decision-making. Cosyne Abstracts 2014, Salt Lake City USA.
