# Supplementary material for: On the Origins of Suboptimality in Human Probabilistic Inference
Source: PLoS Comput Biol. 2014 Jun 19;10(6):e1003661. doi: 10.1371/journal.pcbi.1003661 (PMC4063671; doi:10.1371/journal.pcbi.1003661)
Supplement: Text S3 — Sensorimotor estimation experiment. Methods and results of the additional experiment to estimate the range of subjects' sensorimotor parameters. (PDF) [file pcbi.1003661.s004.pdf]

On the Origins of Suboptimality in Human Probabilistic Inference

L. Acerbi, S. Vijayakumar and D. M. Wolpert

Supporting Text S3 – Sensorimotor estimation experiment

We performed a sensorimotor estimation experiment to obtain an independent measure of subjects' sensorimotor variability (see 'Methods'). The sensorimotor variability includes subjects' noise in determining the location of the cue and projecting it back onto the target line as well as any motor noise in indicating that location. We found that in general the sensorimotor variability was small and had a negligible impact on performance ('Results'). The estimated parameters were used to construct informative priors for the model comparison in the paper ('Informative priors for the model comparison').

Methods

Ten subjects (3 male and 7 female; age range 21–33 years) that had taken part in the main experiment also participated in the control experiment.

The experimental setup had the same layout as the main experiment (see Methods and Figure 1 in the paper), with the following differences: (a) no discrete distribution of targets was shown on screen, only a horizontal target line; (b) in all trials the target was drawn randomly from a uniform distribution whose range covered the width of the active screen window; (c) as usual, half of the trials featured short-distance cues and the other half long-distance cues, but both types of cues had no added noise. In each trial the target was always perfectly above the shown cue, with $x \equiv x_{cue}$.

Subjects performed a short practice session (64 trials) followed by a test session (288 trials). Full performance feedback was provided during both practice and test. Feedback consisted in a visual display of the true position of the target and an integer-valued score that was maximal (10 points) for a perfect 'hit' and decreased rapidly away from the target, according to the following equation:

$$\text{Score}(r, x) = \lfloor 10 \cdot e^{-\frac{(r-x)^2}{2\sigma_{score}^2}} + 0.5 \rfloor$$

where r is the response in the trial, x is the target position, σ_{score} is one-tenth of the cursor diameter ($8.3 \cdot 10^{-3}$ screen units or 2.5 mm) and $\lfloor x \rfloor$ denotes the floor function.

All subjects' datasets for the sensorimotor estimation session are available online in Dataset S1.

Results

Results of the sensorimotor estimation session for all subjects are plotted in Figure 1. The root-mean-squared error (RMSE) of the response with respect to the true target position was on average $(9.3 \pm 0.8) \cdot 10^{-3}$ screen units for long-distance cues and $(5.2 \pm 0.3) \cdot 10^{-3}$ screen units for short-distance cues (mean $\pm$ SE across subjects). In general, the RMSE can be divided in a constant bias term and a variance term, but the bias term was overall small, on average $(0.6 \pm 0.5) \cdot 10^{-3}$ screen units, and not significantly different than zero ($p = 0.26$), which means that the error arose almost entirely from the subject's response variability.

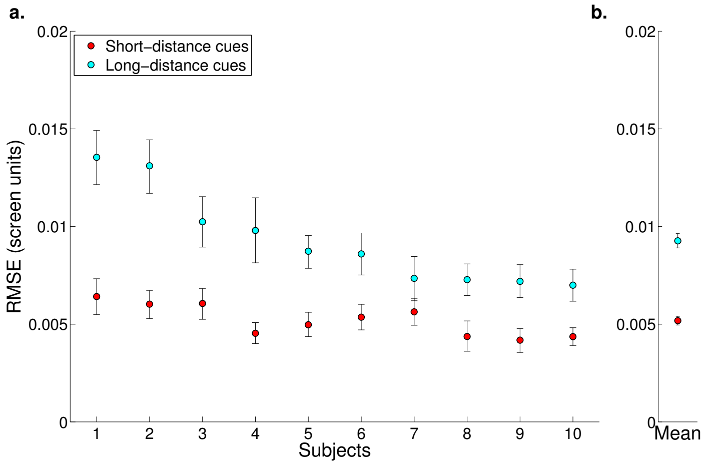

Figure 1. Targeting error for short-distance and long-distance cues. RMSE of the responses, with respect to true target position, for different distance of the cues from the target line, either 'short' (brown dots) or 'long' (blue dots); 0.01 screen units correspond to 3 mm. **a:** Individual RMSE. For visualization, subjects are ordered by average precision. **b:** Mean RMSE, averaged across subjects. In both graphs error bars are 95% confidence intervals computed via bootstrap.

Since subjects knew that the cues were fully informative about the target position, all variability in their responses originated from two sources: sensory noise (error in projecting the cue position on the target line) and motor noise. We assumed that sensory and motor noise were independent and normally distributed, and that sensory variability was proportional to the distance of the cue from the target line (Weber's law). Under these assumptions, variance of subjects' responses was described by the following formula:

$$\sigma^2_{response} = \sigma^2_{motor} + w^2_{sensory} d^2_{cue} \qquad \text{(S1)}$$

where $w_{sensory}$ is Weber's fraction and d_{cue} is the distance of the cue from the target line. Using Eq. S1 we were able to estimate participants' sensorimotor parameters; results are reported in Table 1.

Parameter	Description	Mean $\pm$ Std (screen units)	Mean $\pm$ Std (mm)
σ_{motor}	Motor noise	$(3.6 \pm 1.1) \cdot 10^{-3}$	1.1 ± 0.3
Σ_{low}	Sensory noise (short cues)	$(3.5 \pm 1.1) \cdot 10^{-3}$	1.1 ± 0.3
Σ_{high}	Sensory noise (long cues)	$(8.1 \pm 2.6) \cdot 10^{-3}$	2.4 ± 0.8

Table 1. Average estimated sensorimotor parameters. Group-average estimated motor and sensory noise parameters. Estimates were obtained from the data through Eq. S1.

The estimated parameters in Table 1 allowed us to assess the typical impact of realistic values of sensorimotor noise on subjects' performance. First, we computed the performance of the optimal ideal observer model with added realistic noise. In order to do so, we generated 1000 subjects by sampling from the distribution of estimated sensorimotor parameters and we then simulated their behavior on our subjects' datasets according to the optimal observer model. We found an average optimality index of 0.997 ± 0.001 which is empirically indistinguishable from one. The difference in performance induced by the sensorimotor noise was analogously negligible for the simulations of other ideal observer models, such

as the 'prior-only' or 'cue-only' models (see Figure 5 in the paper). These results show that motor and sensory noise had a very limited impact on subjects' performance.

Informative priors for the model comparison

The pooled estimated parameters summarized in Table 1 were used to construct informative priors for the motor and sensory parameters that were applied in our model comparison (see paper and Text S1). Bootstrapped parameters were fit with log-normal distributions with log-scale μ and shape parameter σ (which correspond to mean and SD in log space; see Figure 2). The resulting parameters of the priors were $\mu = \log 3.4 \cdot 10^{-3}$ screen units, $\sigma = 0.38$ for σ_{motor}; and $\mu = \log 7.7 \cdot 10^{-3}$ screen units, $\sigma = 0.32$ for Σ_{high}. The prior on σ_{motor} was used in all observer models, whereas the prior on Σ_{high} was used only in the observer models with sensory noise (model factor S).

Using an independent experiment to construct informative priors can be thought of as a 'soft' generalization of the typical procedure that consists in directly applying independently estimated parameters to an observer model [1]. In that case, the constructed priors are delta functions on point estimates of the subjects' parameters. Here, instead, pooled measured parameters were used to compute distributions that represent realistic values for the model parameters in our task (that is, informative priors).

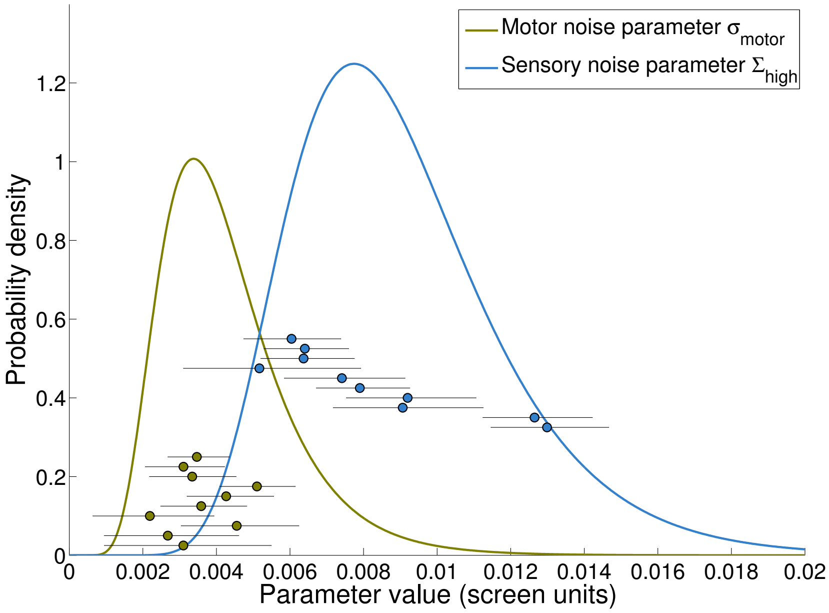

Figure 2. Priors over sensorimotor parameters. The experimental estimates of individual parameters for motor noise (brown dots) and sensory noise (purple dots) are used to construct informative log-normal priors for σ_{motor} (brown line) and Σ_{high} (purple line) in the main experiment. Error bars are 95% confidence intervals, computed via bootstrap.

References

1. Tassinari H, Hudson T, Landy M (2006) Combining priors and noisy visual cues in a rapid pointing task. J Neurosci 26: 10154–10163.
